# Visible-light photocatalytic synthesis of imidazole-2-thiones in water using cobalt phthalocyanine

**DOI:** 10.1039/d6ra03480b

**Published:** 2026-05-22

**Authors:** Amin Arman, Najmeh Nowrouzi, Mohammad Abbasi, Jan Janczak

**Affiliations:** a Department of Chemistry, Faculty of Nano and Bio Science and Technology, Persian Gulf University Bushehr 75169 Iran nowrouzi@pgu.ac.ir; b Institute of Low Temperature and Structure Research, Polish Academy of Sciences P.O. Box 1410, Okolna 2 str. 50-950 Wroclaw Poland

## Abstract

A sustainable and efficient protocol for the synthesis of imidazole-2-thione derivatives is herein reported. The method relies on the reaction of imidazolium salts with sodium sulfide (Na_2_S) in water at room temperature under visible-light irradiation. In this transformation, cobalt phthalocyanine (CoPc) serves as an inexpensive and robust catalyst, while ambient air acts as a clean and environmentally benign terminal oxidant. Operational simplicity, straightforward work-up, and excellent product yields highlight the practicality of this environmentally friendly methodology, making it an attractive approach for the green synthesis of imidazole-2-thiones.

## Abstract 2

A sustainable and efficient protocol for the synthesis of imidazole-2-thione derivatives is herein reported.

## Graphical abstract

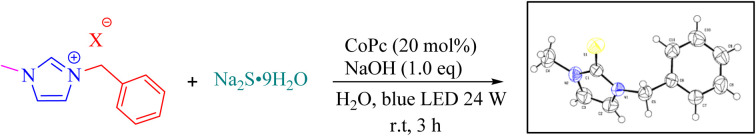

## Introduction

In recent years, visible-light-mediated organic transformations have attracted significant attention and have emerged as powerful, reliable, and environmentally benign synthetic methodologies.^1–3^ Compared with traditional thermal or transition-metal-catalyzed processes, visible-light-promoted single-electron transfer (SET) or electron donor–acceptor (EDA) excitation enables the efficient and selective generation of reactive radical intermediates.^4–9^

These strategies have been widely applied to carbon–heteroatom bond formation, particularly C–S coupling reactions, which are essential for the synthesis of pharmaceuticals and functional materials. Considerable progress has recently been made in developing visible-light-driven methods for constructing C–S bonds.^10–16^ Liu and co-workers developed a visible-light-driven, mild cross-coupling protocol for thiols and aryl halides, enabling the formation of thioethers.^17^ In another study, Kibriya and co-workers described a visible-light-promoted oxidative coupling of thiols with arylhydrazines to generate diaryl sulfides.^18^ In 2019, Li *et al.* developed a multicomponent reaction for the synthesis of *S*-aryl dithiocarbamates under visible-light irradiation.^19^ Subsequently, Uchikura *et al.* introduced a visible-light-driven C(sp^3^)–S bond-forming protocol operating through a dual EDA–SET and hydrogen atom transfer (HAT) mechanism to access sulfide products.^20^ Later, Wang and co-workers reported an intramolecular C(sp^2^)–S cyclization of *ortho*-halothiobenzanilides to benzothiazoles under visible light.^21^ Most recently, our group developed a novel strategy for the synthesis of thioethers and sulfur-bridged bis-enamiones using elemental sulfur as a sustainable sulfur source under visible-light irradiation.^22,23^ Collectively, these developments highlight the growing relevance of visible light as a sustainable and powerful platform for the selective construction of carbon–sulfur bonds.

Imidazole-2-thiones represent an important class of sulfur-containing heterocycles owing to their broad pharmacological activities and diverse industrial applications. For example, 1-methylimidazole-2-thione is clinically employed in the treatment of various diseases,^24,25^ while imidazole-2-thione C-nucleosides serve as versatile synthetic intermediates for the preparation of azido- and fluoro-nucleosides, both recognized for their notable anti-HIV activity.^26^ Moreover, they have been investigated for antimicrobial, antifungal, and antiviral properties, reflecting broad pharmacological potential.^27^ Beyond biology, the 2-thione functionality enables coordination with metals, useful in synthesis and catalysis, and supports construction of more complex heterocycles, highlighting their utility in organic synthesis and material chemistry.^28^

**Scheme 1:**
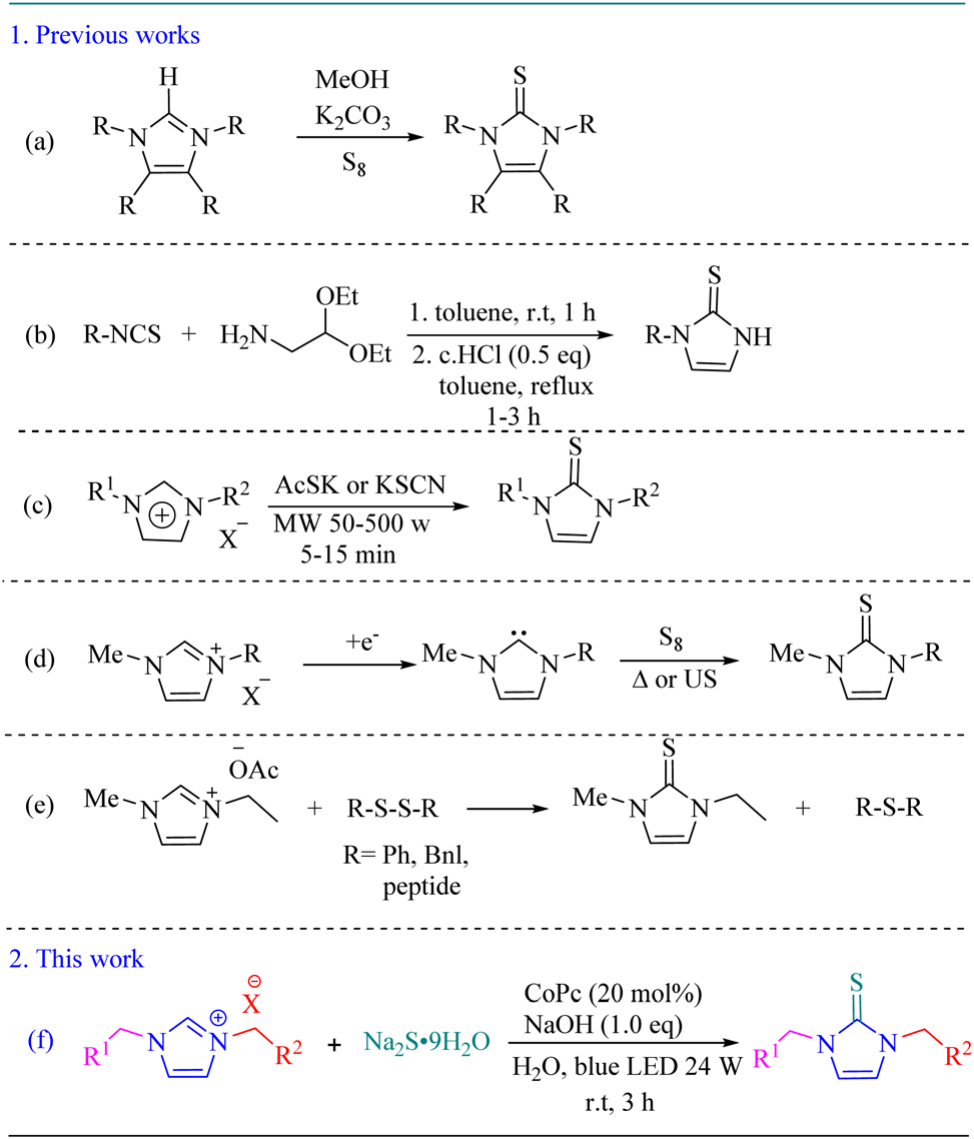
Synthesis of imidazole-2-thiones.

A number of strategies have been developed for the synthesis of imidazole-2-thiones (Scheme 1). The most common procedure to access imidazole-2-thiones involves the reaction between imidazolium salts and elemental sulfur (S_8_) in the presence of a base (Scheme 1a).^29–31^ Furthermore, 1-substituted imidazole-2-thione derivatives have been prepared by reacting isothiocyanate with amino acetals in toluene at 110 °C for 1–3 h (Scheme 1b).^32^ In 2007, Tao and co-workers reported a microwave-assisted, solvent-free protocol for the synthesis of imidazole-2-thiones from imidazolium salts and metal thioacetate or thiocyanate (Scheme 1c).^33^ Feroci and colleagues synthesized 1,3-dialkylimidazole-2-thiones *via* electrochemical reduction of imidazolium salts to generate the corresponding *N*-heterocyclic carbenes, followed by reaction with elemental sulfur under ultrasound irradiation (Scheme 1d).^34^ In another study, Baumruck *et al.* obtained 1-ethyl-3-methylimidazole-2-thione *via* the reaction of corresponding imidazolium acetate with sulfur-containing compounds and peptides (Scheme 1e).^35^

Although significant advances have been achieved in visible-light-mediated C–S bond formation, most reported methods mainly focus on the synthesis of thioethers and related sulfur-containing compounds. In contrast, reports concerning the visible-light-driven synthesis of imidazole-2-thiones under environmentally benign aqueous conditions remain very limited. Therefore, the development of a simple and efficient protocol for the synthesis of imidazole-2-thiones in water under visible-light irradiation using an inexpensive catalyst remains highly desirable. Herein, we report an efficient and straightforward visible-light-driven protocol for the synthesis of imidazole-2-thione derivatives through the reaction of imidazolium salts with sodium sulfide (Na_2_S) at room temperature, using cobalt phthalocyanine as a low-cost and readily available photocatalyst and ambient air as the terminal oxidant (Scheme 1f).

## Results and discussion

We initiated our study using 3-benzyl-1-methyl-1*H*-imidazole-3-ium chloride (1) and sodium sulfide (2) as model substrates, employing NaOH (1.0 equiv.) in DMF at 80 °C for 12 h. Encouragingly, the oxidative coupling product (3a) was isolated in 56% yield (Table 1, entry 1). Motivated by this outcome, further control experiments were performed to optimize the reaction. First, the effect of various solvents, including DMSO, EtOH, CH_3_CN, 1,4-dioxane, and H_2_O, was investigated (Table 1, entries 2–6). Among these, DMSO and H_2_O proved most effective, whereas EtOH, CH_3_CN, and 1,4-dioxane gave lower yields. Notably, the reaction did not proceed efficiently under solvent-free conditions (Table 1, entry 7). So, H_2_O was ultimately selected due to its environmentally benign, inexpensive, readily available, and non-hazardous nature. The reaction was found to be sensitive to the choice of base (Table 1, entries 8–13). KOH and NaOH provided the highest yields, while other bases such as NaHCO_3_, Na_2_CO_3_, K_2_CO_3_, and Et_3_N resulted in diminished product formation. In the absence of a base, the yield was significantly lower (Table 1, entry 14). Based on these results, NaOH was selected as the base for subsequent reactions. Importantly, the addition of 20 mol% cobalt phthalocyanine (CoPc) gave the desired product in an outstanding 92% yield (Table 1, entry 15), indicating the necessity of a photocatalyst. Subsequently, the reaction conditions were further investigated to evaluate the effects of temperature and visible-light irradiation. When the reaction was performed at room temperature for 12 h, product 3a was obtained in only 34% yield (Table 1, entry 16). In sharp contrast, irradiation with a 24 W blue LED dramatically improved the reaction efficiency, affording the desired product in an excellent 93% yield within only 3 h (Table 1, entry 17). Encouraged by this result, the influence of different visible-light sources was subsequently examined. As summarized in Table 1 (entries 18–20), red, green, and white LEDs furnished lower yields compared to blue LED irradiation, indicating that blue LED irradiation provides the most efficient conditions among the tested light sources. Moreover, under otherwise identical dark conditions, merely a trace amount of the product was detected after 3 h, highlighting the sluggish nature of the transformation in the absence of light (Table 1, entry 21). These observations confirm the crucial role of visible-light irradiation in promoting the reaction. Finally, the loading of the photocatalyst was further optimized under the established optimal conditions. When the amount of CoPc was decreased to 10 mol%, a lower yield was obtained, whereas increasing the amount of CoPc to 30 mol% resulted in no further improvement in yield (Table 1, entries 22, 23). The conditions outlined in entry 17 were identified as optimal for the synthesis of imidazole-2-thione derivatives.

**Table 1:** Screening of reaction conditions

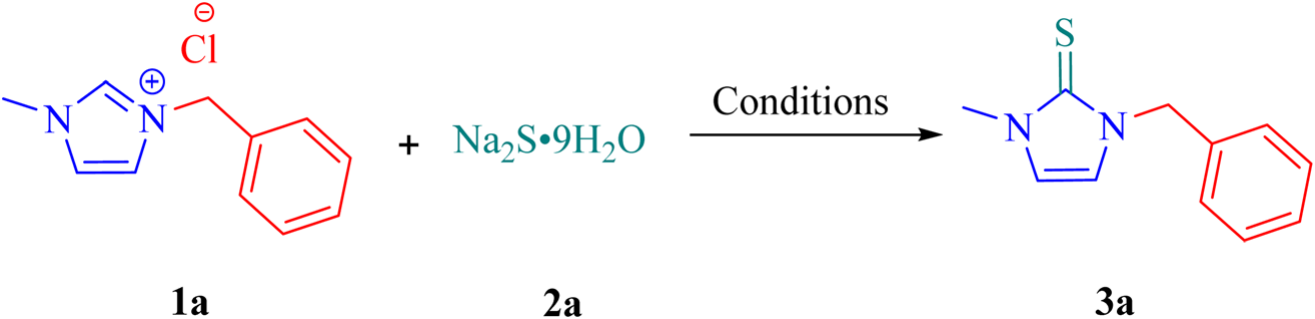						
Entry	Solvent	Base (1.0 mmol)	Oxidant/photocatalyst	Temp/light	Time (h)	Yield^a^ %
1	DMF	NaOH	Air/–	80 °C	12	56
2	DMSO	NaOH	Air/–	80 °C	12	64
3	EtOH	NaOH	Air/–	80 °C	12	52
4	CH_3_CN	NaOH	Air/–	80 °C	12	38
5	1,4-Dioxane	NaOH	Air/–	80 °C	12	49
6	H_2_O	NaOH	Air/–	80 °C	12	65
7	Solvent free	NaOH	Air/–	80 °C	12	47
8	H_2_O	KOH	Air/–	80 °C	12	65
9	H_2_O	NaHCO_3_	Air/–	80 °C	12	47
10	H_2_O	Na_2_CO_3_	Air/–	80 °C	12	51
11	H_2_O	K_2_CO_3_	Air/–	80 °C	12	57
12	H_2_O	NaOAc	Air/–	80 °C	12	55
13	H_2_O	Et_3_N	Air/–	80 °C	12	33
14	H_2_O	—	Air/–	80 °C	12	21
15	H_2_O	NaOH	Air/CoPc (20 mol%)	80 °C	12	92
16	H_2_O	NaOH	Air/CoPc (20 mol%)	Rt, no LED	12	34
**17**	**H**_**2**_ **O**	**NaOH**	**Air/CoPc (20 mol%)**	**24 W blue LED**	**3**	**93**
18	H_2_O	NaOH	Air/CoPc (20 mol%)	24 W green LED	3	79
19	H_2_O	NaOH	Air/CoPc (20 mol%)	24 W red LED	3	70
20	H_2_O	NaOH	Air/CoPc (20 mol%)	24 W white LED	3	87
21	H_2_O	NaOH	Air/CoPc (20 mol%)	Rt, dark	3	Trace
22	H_2_O	NaOH	Air/CoPc (10 mol%)	24 W blue LED	3	56
23	H_2_O	NaOH	Air/CoPc (30 mol%)	24 W blue LED	3	93

a Reaction conditions: 1a (1.0 mmol), 2a (1.2 mmol), CoPc (20 mol%), base (1.0 mmol), solvent (1 mL), and 24 W blue LED for 3 h.

With the optimized conditions established, a series of 1,3-disubstituted imidazolium salts prepared *via* the reaction of imidazole or 1-methylimidazole with benzyl, alkenyl, and alkyl halides^36–40^ were employed for the synthesis of imidazole-2-thiones, as summarized in Table 2.

**Table 2:** Synthesis of imidazole-2-thiones^a^

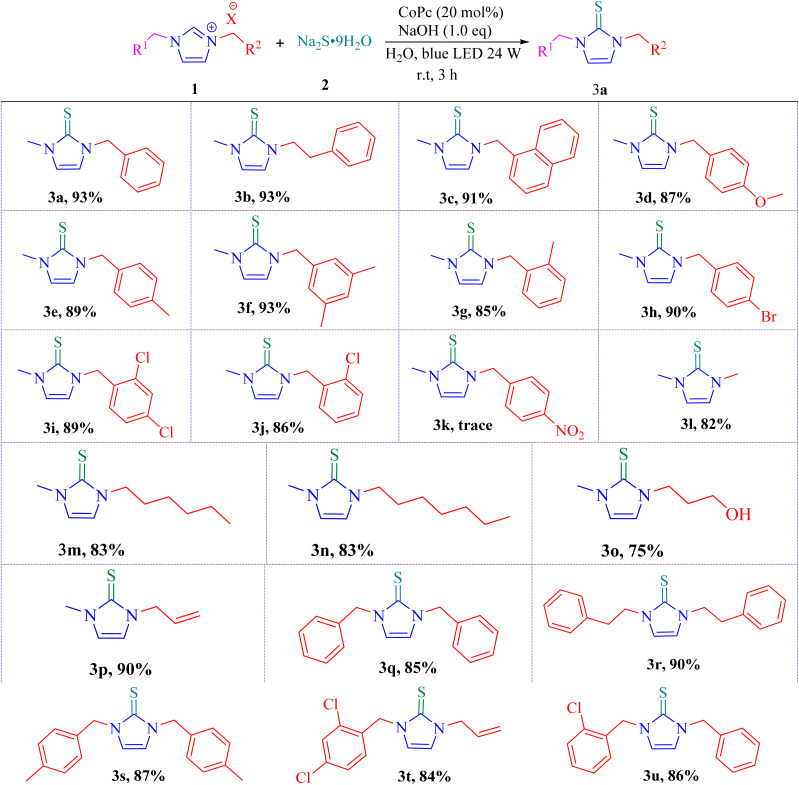

a Reaction conditions: 1 (1.0 mmol), 2 (1.2 mmol), NaOH (1.0 mmol), CoPc (20 mol%), H_2_O (1 mL) under a 24 W blue LED irradiation for 3 h.

As shown in Table 2, the reaction of 1-benzyl-3-methyl-, 1-methyl-3-phenethyl-, and 1-methyl-3-(naphthalen-1-ylmethyl) imidazolium salts with sodium sulfide afforded the corresponding products 3a–3c in excellent yields. Imidazolium salts bearing aryl rings with electron-donating groups (*p*-OMe, *p*-Me, 3,4-dimethyl, and *o*-Me) also reacted efficiently, providing the desired products 3d–3g in high yields. Salts containing weak electron-withdrawing groups (*p*-Br, 2,3-dichloro, *o*-Cl) similarly underwent smooth reactions, giving 3h–3j under the optimized conditions. In contrast, the imidazolium salt bearing an aryl ring substituted with a strongly electron-withdrawing *p*-nitro group afforded only a trace amount of the desired product 3k. In this case, TLC analysis indicated the formation of several unidentified by-products, which significantly reduced the yield of the main product. The diminished efficiency can reasonably be attributed to the strong electron-withdrawing character and oxidative nature of the nitro group. Under the reaction conditions, this substituent may promote competing oxidative pathways or side reactions, leading to the formation of undesired by-products and suppressing the generation of the target imidazole-2-thione. Furthermore, 1,3-dialkyl imidazolium salts, such as 1,3-dimethyl-, 3-hexyl-1-methyl-, and 3-heptyl-1-methyl-substituted derivatives, reacted well to furnish imidazole-2-thiones 3l–3n in good yields. Notably, 3-(3-hydroxypropyl)-1-methyl-1*H*-imidazole-3-ium chloride afforded the corresponding product 3o in 75% yield. This result indicates that the presence of a free hydroxyl group is tolerated under the optimized reaction conditions and does not interfere with product formation. 3-Allyl-1-methyl-imidazolium salt, which contains a double bond, proved to be a suitable substrate, affording the corresponding product 3p in 90% yield. To further explore the substrate scope, symmetrical imidazolium salts, including 1,3-dibenzyl-, 1,3-diphenethyl-, and 1,3-bis(4-methylbenzyl)-substituted imidazolium salts, were employed and afforded the corresponding imidazole-2-thiones 3q–3s in excellent yields. Additionally, unsymmetrical imidazolium salts lacking methyl substituents also exhibited high reactivity, furnishing products 3t and 3u in 84% and 86% yields, respectively.

**Fig. 1:**
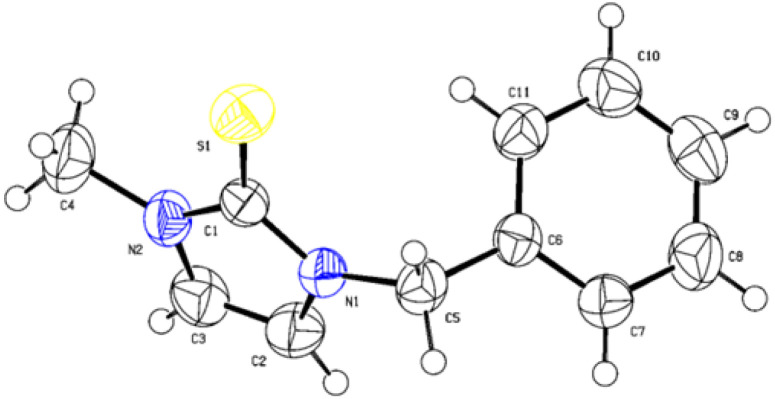
Single crystal X-ray structure of (3a) (CCDC 2528299).

The structures of all reaction products were confirmed by NMR spectroscopy, and the full spectral data are provided in the SI. Additionally, the molecular structure of product 3a was definitively confirmed by single-crystal X-ray diffraction (Fig. 1), further validating the proposed structure and confirming the reliability of our synthetic method.

**Scheme 2:**
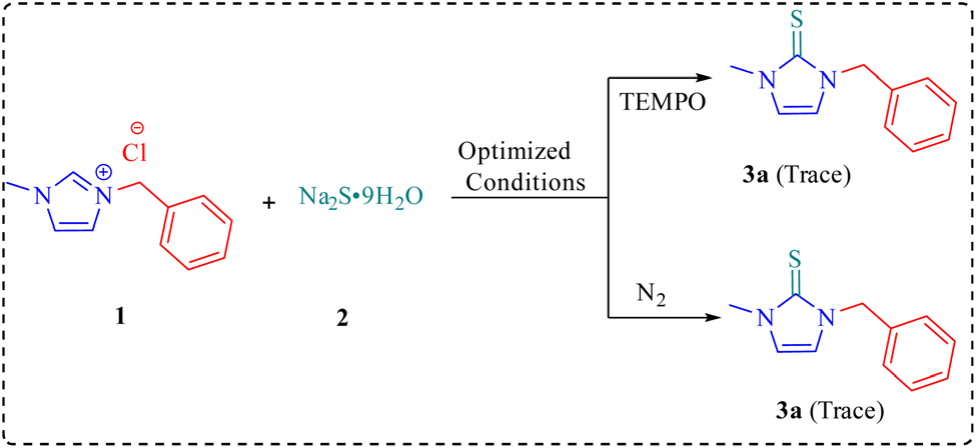
Control experiments.

**Scheme 3:**
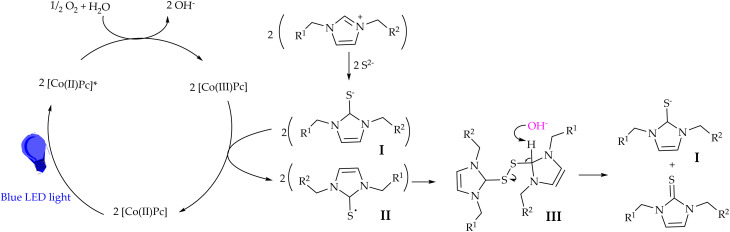
Proposed mechanism.

To probe the formation of imidazole-2-thione 3, some control experiments were performed (Scheme 2). Notably, upon addition of TEMPO as a radical scavenger under the optimized conditions, only a trace amount of product 3a was obtained, suggesting the involvement of a radical pathway. In another experiment, the role of molecular oxygen for this transformation was confirmed by conducting the reaction under nitrogen atmosphere, which also yielded a trace amount of the product 3a. Accordingly, the following mechanism is proposed for this transformation (Scheme 3).

The catalytic cycle is initiated by blue LED irradiation, which excites Co(ii) phthalocyanine (Co(ii)*Pc*) to its photoexcited state (Co(ii)Pc*). The excited catalyst undergoes single-electron transfer with molecular oxygen, generating Co(iii)*Pc* and reduced oxygen species, while water participates in the formation of hydroxide ions. Molecular oxygen thus serves as the terminal and environmentally benign oxidant. Meanwhile, the imidazolium salt reacts with sulfide ions to form a sulfur-containing intermediate I. This intermediate is then oxidized by Co(iii)*Pc* to produce a radical species II. Two such radicals undergo radical–radical coupling to form intermediate III, followed by deprotonation, leading to the formation of the imidazole-2-thione product. Notably, intermediate I is also regenerated during the process, allowing it to re-enter the catalytic cycle. The cobalt catalyst is simultaneously reduced back to Co(ii)*Pc*, thereby completing the photocatalytic cycle.

## Conclusion

In conclusion, we have developed a visible-light-induced oxidative C–S coupling strategy between imidazolium salts and sodium sulfide, enabling the efficient synthesis of both symmetrical and unsymmetrical imidazole-2-thiones. A wide range of imidazole-2-thiones was obtained in high yields at ambient temperature under visible-light irradiation. The use of cobalt phthalocyanine as an inexpensive and accessible catalyst, together with water as a green solvent and ambient air as a benign terminal oxidant, makes this protocol operationally simple and synthetically practical. We believe that this method offers significant potential and utility for future applications in organic synthesis.

## Experimental section

### General procedure for the synthesis of imidazole-2-thiones

A mixture of the imidazolium salt (1.0 mmol), Na_2_S·9H_2_O (1.2 mmol), cobalt phthalocyanine (20 mol%), and sodium hydroxide (1.0 mmol) in H_2_O (1 mL) was stirred for 3 hours while being irradiated with a 24 W blue LED light source. After TLC-confirmed completion, the mixture was extracted with ethyl acetate (3 × 1 mL) followed by removal of the solvent under reduced pressure. Purification of the solid products was accomplished by recrystallization from *n*-hexane/ethyl acetate (10 : 2), Whereas, Column chromatography on silica gel with *n*-hexane/ethyl acetate (10 : 5) was employed to purify the liquid products 3m–3p.

## Author contributions

Amin Arman: investigation, methodology, data curation, writing – original draft; Najmeh Nowrouzi: project administration, writing – review & editing; Mohammad Abbasi: data analysis, supervision; Jan Janczak: X-ray spectra acquisition.

## Conflicts of interest

There are no conflicts to declare.

## Supplementary Material

RA-016-D6RA03480B-s001

RA-016-D6RA03480B-s002

## Data Availability

The data supporting this article have been included as part of the supplementary information (SI). Supplementary information is available. See DOI: https://doi.org/10.1039/d6ra03480b. CCDC 2528299 contains the supplementary crystallographic data for this paper.^41^
