# Supplementary material for: Visible-light photocatalytic synthesis of imidazole-2-thiones in water using cobalt phthalocyanine
Source: RSC Adv. 2026 May 22;16(30):27878–83. doi: 10.1039/d6ra03480b (PMC13202418; doi:10.1039/d6ra03480b)
Supplement: RA-016-D6RA03480B-s001 [file RA-016-D6RA03480B-s001.pdf]

Supplementary Information (SI) for RSC Advances.
This journal is © The Royal Society of Chemistry 2026

Supporting Information for

Visible-Light Photocatalytic Synthesis of Imidazole-2-Thiones in Water using Cobalt Phthalocyanine

Amin Arman,[a] Najmeh Nowrouzi,*[a] Mohammad Abbasi,[a] Jan Janczak[b]

[a] Department of Chemistry, Faculty of Nano and Bio Science and Technology, Persian Gulf University, Bushehr 75169 Iran

[b] Institute of Low Temperature and Structure Research, Polish Academy of Sciences, P.O. Box 1410, Okolna 2 str., 50-950 Wroclaw, Poland

Email: nowrouzi@pgu.ac.ir

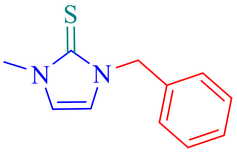

1-benzyl-3-methyl-1,3-dihydro-2H-imidazole-2-thione (Table 2, 3a). ^{1}H-NMR (300 MHz, $CDCl_3$) δ (ppm): 3.66 (s, 3H, CH_3), 5.27 (s, 2H, CH_2), 6.59 (d, J = 3.2 Hz, 1H, CH), 6.69 (d, J = 3.2 Hz, 1H, CH), 7.29-7.37 (m, 5H, Ar). ^{13}C-NMR (75 MHz, $CDCl_3$) δ (ppm): 35.3, 51.4, 116.4, 118.0, 128.2, 128.3, 128.9, 135.9, 162.9.

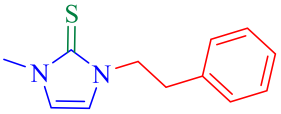

1-methyl-3-phenethyl-1,3-dihydro-2H-imidazole-2-thione (Table 2, 3b). ^{1}H-NMR (400 MHz, $CDCl_3$) δ (ppm): 3.09 (t, J=7.6 Hz, 2H, CH_2), 3.62 (s, 3H, CH_3), 4.27 (t, J=7.6 Hz, 2H, CH_2), 6.38 (d, J=2.0 Hz, 1H, CH), 6.59 (d, J=2.0 Hz, 1H, CH), 7.19-7.26 (m, 3H, Ar), 7.28-7.32 (m, 2H, Ar). ^{13}C-NMR (100 MHz, $CDCl_3$) δ (ppm): 34.9, 35.1, 49.6, 117.1, 117.3, 126.7, 128.6, 129.0, 137.9, 161.9.

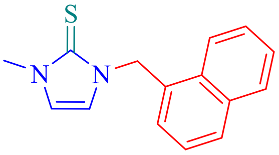

1-methyl-3-(naphthalen-1-ylmethyl)-1,3-dihydro-2H-imidazole-2-thione (Table 2, 3c). ^{1}H-NMR (400 MHz, $CDCl_3$) δ (ppm): 3.67 (s, 3H, CH_3), 5.64 (s, 2H, CH_2), 6.35 (d, J=2.4 Hz, 1H, CH), 6.58 (d, J=2.4 Hz, 1H, CH), 7.44 (dd, J_1=7.2, J_2=1.6 Hz, 1H, Ar), 7.47 (d, J=7.6 Hz, 1H, Ar), 7.49-7.57 (m, 2H, Ar), 7.87-7.90 (m, 2H, Ar), 8.01(dd, J_1=7.6, J_2=2.4 Hz, 1H, Ar). ^{13}C-NMR (100 MHz, $CDCl_3$) δ (ppm): 35.2, 49.8, 116.0, 117.9, 123.9, 125.3, 126.3, 127.1, 128.0, 128.7, 129.5, 131.2, 131.4, 133.9, 162.3.

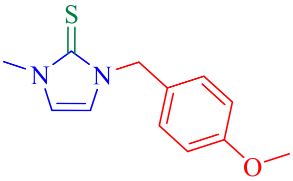

1-(4-methoxybenzyl)-3-methyl-1,3-dihydro-2H-imidazole-2-thione (Table 2, 3d). ^{1}H-NMR (400 MHz, $CDCl_3$) δ (ppm): 3.62 (s, 3H, CH_3), 3.78 (s, 3H, OCH_3), 5.16 (s, 2H, CH_2), 6.54 (d, J=2.4 Hz, 1H, CH), 6.65 (d, J=2.4 Hz, 1H, CH), 6.86 (d, J=8.4 Hz, 2H, Ar), 7.26 (d, J=8.4 Hz, 2H, Ar). ^{13}C-NMR (100 MHz, $CDCl_3$) δ (ppm): 35.2, 50.9, 55.3, 114.2, 116.2, 117.9, 127.9, 129.9, 159.5, 162.5.

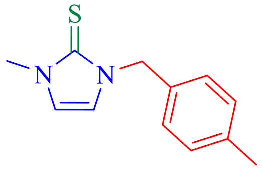

1-methyl-3-(4-methylbenzyl)-1,3-dihydro-2H-imidazole-2-thione (Table 2, 3e). ^{1}H-NMR (400 MHz, $CDCl_3$) δ (ppm): 2.35 (s, 3H, CH_3), 3.65 (s, 3H, CH_3), 5.21 (s, 2H, CH_2), 6.56 (d, *J*=2.4 Hz, 1H, CH), 6.66 (d, *J*=2.4 Hz, 1H, CH), 7.16 (d, *J*=8.0 Hz, 2H, Ar), 7.23 (d, *J*=8.0 Hz, 2H, Ar). ^{13}C-NMR (100 MHz, $CDCl_3$) δ (ppm): 21.2, 35.2, 51.1, 116.3, 118.0, 128.3, 129.5, 132.8, 137.9, 162.6.

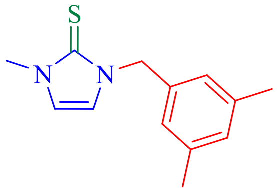

1-(3,5-dimethylbenzyl)-3-methyl-1,3-dihydro-2H-imidazole-2-thione (Table 2, 3f). ^{1}H-NMR (400 MHz, $CDCl_3$) δ (ppm): 2.30 (s, 6H, CH_3), 3.66 (s, 3H, CH_3), 5.18 (s, 2H, CH_2), 6.57 (d, *J*=2.4 Hz, 1H, CH), 6.67 (d, *J*=2.4 Hz, 1H, CH), 6.92 (s, 2H, Ar), 6.95 (s, 1H, Ar). ^{13}C-NMR (100 MHz, $CDCl_3$) δ (ppm): 21.3, 35.2, 51.3, 116.4, 117.9, 126.1, 129.8, 135.7, 138.5, 162.7.

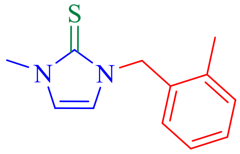

1-methyl-3-(2-methylbenzyl)-1,3-dihydro-2H-imidazole-2-thione (Table 2, 3g). ^{1}H-NMR (400 MHz, $CDCl_3$) δ (ppm): 2.26 (s, 3H, CH_3), 3.66 (s, 3H, CH_3), 5.19 (s, 2H, CH_2), 6.38 (d, *J*=2.4 Hz, 1H, CH), 6.67 (d, *J*=2.4 Hz, 1H, CH), 7.13 (d, *J*=7.2 Hz, 1H, Ar), 7.18-7.29 (m, 3H, Ar). ^{13}C-NMR (100 MHz, $CDCl_3$) δ (ppm): 19.3, 35.2, 49.9, 116.0, 117.9, 126.4, 128.5, 129.4, 130.8, 133.4, 137.0, 162.6.

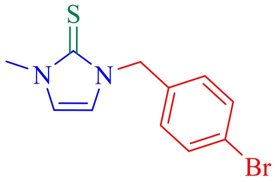

1-(4-bromobenzyl)-3-methyl-1,3-dihydro-2H-imidazole-2-thione (Table 2, 3h). ^{1}H-NMR (400 MHz, $CDCl_3$) δ (ppm): 3.65 (s, 3H, CH_3), 5.22 (s, 2H, CH_2), 6.59 (d, *J*=2.4 Hz, 1H, CH), 6.69 (d, *J*=2.4 Hz, 1H, CH), 7.21 (d, *J*=8.4 Hz, 2H, Ar), 7.47 (d, *J*=8.4 Hz, 2H, Ar). ^{13}C-NMR (100 MHz, $CDCl_3$) δ (ppm): 35.3, 50.7, 116.2, 118.2, 122.2, 129.9, 132.0, 135.0, 163.1.

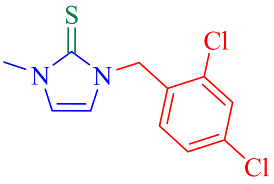

1-(2,4-dichlorobenzyl)-3-methyl-1,3-dihydro-2H-imidazole-2-thione (Table 2, 3i). ^{1}H-NMR (400 MHz, $CDCl_3$) δ (ppm): 3.66 (s, 3H, CH_3), 5.36 (s, 2H, CH_2), 6.71(d, J=2.4 Hz, 1H, CH), 6.72 (d, J=2.4 Hz, 1H, CH), 7.23 (dd, J_1=8.4, J_2=2.0 Hz, 1H, Ar), 7.35 (d, J=8.4 Hz, 1H, Ar), 7.44 (d, J=2.0 Hz, 1H, Ar). ^{13}C-NMR (100 MHz, $CDCl_3$) δ (ppm): 35.4, 48.1, 116.6, 118.2, 127.6, 129.5, 131.4, 132.2, 134.0, 134.7, 163.3.

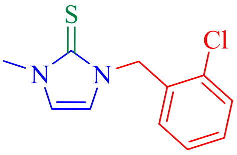

1-(2-chlorobenzyl)-3-methyl-1,3-dihydro-2H-imidazole-2-thione (Table 2, 3j). ^{1}H-NMR (400 MHz, $CDCl_3$) δ (ppm): 3.66 (s, 3H, CH_3), 5.39 (s, 2H, CH_2), 6.68 (d, J=2.4 Hz, 1H, CH), 6.70 (d, J=2.4 Hz, 1H, CH), 7.22-7.30 (m, 2H, Ar), 7.35 (dd, J_1=6.8, J_2=2.4 Hz, 1H, Ar), 7.41(dd, J_1=7.2, J_2=2.0 Hz, 1H, Ar), ^{13}C-NMR (100 MHz, $CDCl_3$) δ (ppm): 35.3, 48.7, 116.6, 118.0, 127.4, 129.6, 129.7, 130.5, 133.4, 133.5, 163.2.

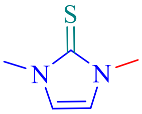

1,3-dimethyl-1,3-dihydro-2H-imidazole-2-thione (Table 2, 3l). ^{1}H-NMR (400 MHz, $CDCl_3$) δ (ppm): 3.63 (s, 6H, CH_3), 6.69 (s, 2H, CH). ^{13}C-NMR (100 MHz, $CDCl_3$) δ (ppm): 35.3, 117.6, 163.1.

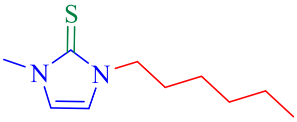

1-hexyl-3-methyl-1,3-dihydro-2H-imidazole-2-thione (Table 2, 3m). ^{1}H-NMR (400 MHz, $CDCl_3$) δ (ppm): 0.83-0.86 (m, 3H, CH_3), 1.25-1.35 (m, 6H, CH_2), 169-177 (m, 2H, CH_2), 3.58 (s, 3H, CH_3), 3.97-4.01 (m, 2H, CH_2), 6.67 (d, J=2.4 Hz, 1H, CH), 6.68 (d, J=2.4 Hz, 1H, CH). ^{13}C-NMR (100 MHz, $CDCl_3$) δ (ppm): 14.0, 22.5, 26.2, 28.7, 31.3, 35.0, 48.1, 116.5, 117.6, 162.0.

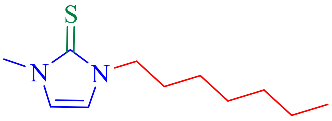

1-heptyl-3-methyl-1,3-dihydro-2H-imidazole-2-thione (Table 2, 3n). ^{1}H-NMR (400 MHz, $CDCl_3$) δ (ppm): 0.86 (t, J=6.8 Hz, 3H, CH_3), 1.22-1.34 (m, 8H, CH_2), 1.71-1.78 (m, 2H, CH_2), 3.60 (s, 3H, CH_3), 4.00 (t, J=7.6 Hz, 2H, CH_2), 6.67 (d, J=2.4 Hz, 1H, CH), 6.68 (d, J=2.4 Hz, 1H, CH). ^{13}C-NMR (100 MHz, $CDCl_3$) δ (ppm): 14.1, 22.5, 26.5, 28.9, 31.7, 35.0, 48.1, 116.5, 117.6, 162.0.

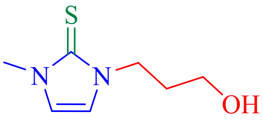

1-(3-hydroxypropyl)-3-methyl-1,3-dihydro-2H-imidazole-2-thione (Table 2, 3o). ^{1}H-NMR (400 MHz, $CDCl_3$) δ (ppm): 1.91 (quint, J=6.0 Hz, 2H, CH_2), 3.50 (t, J=6.0 Hz, 2H, CH_2), 3.64 (s, 3H, CH_3), 4.25 (t, J=6.0 Hz, 2H, CH_2), 6.73 (d, J=2.4 Hz, 1H, CH), 6.76 (d, J=2.4 Hz, 1H, CH), 7.29 (s, 1H, OH). ^{13}C-NMR (100 MHz, $CDCl_3$) δ (ppm): 32.6, 35.3, 43.9, 57.0, 116.7, 118.5, 162.2.

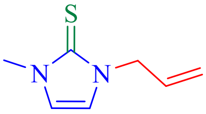

1-allyl-3-methyl-1,3-dihydro-2H-imidazole-2-thione (Table 2, 3p). ^{1}H-NMR (400 MHz, $CDCl_3$) δ (ppm): 3.63 (s, 3H, CH_3), 4.68 (dt, J_1=6.0, J_2=1.6 Hz, 2H, CH_2), 5.21-5.30 (m, 2H, CH_2), 5.88-5.98 (m, 1H, CH), 6.69 (d, J=2.4 Hz, 1H, CH), 6.71 (d, J=2.4 Hz, 1H, CH). ^{13}C-NMR (100 MHz, $CDCl_3$) δ (ppm): 35.2, 50.3, 116.2, 117.8, 119.1, 131.9, 162.5.

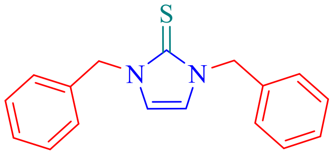

1,3-dibenzyl-1,3-dihydro-2H-imidazole-2-thione (Table 2, 3q). ^{1}H-NMR (400 MHz, $CDCl_3$) δ (ppm): 5.31 (s, 4H, CH_2), 6.56 (s, 2H, CH), 7.29-7.38 (m, 10H, Ar). ^{13}C-NMR (100 MHz, $CDCl_3$) δ (ppm): 51.4, 116.8, 128.2, 128.4, 128.9, 135.8, 163.2.

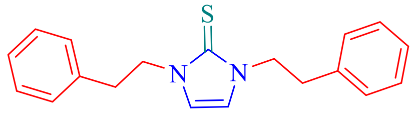

1,3-diphenethyl-1,3-dihydro-2H-imidazole-2-thione (Table 2, 3r). ^{1}H-NMR (400 MHz, $CDCl_3$) δ (ppm): 3.10-3.14 (m, 4H, CH_2), 4.28-4.32 (m, 4H, CH_2), 6.25 (s, 2H, CH), 7.19-7.34 (m, 10H, Ar). ^{13}C-NMR (100 MHz, $CDCl_3$) δ (ppm): 34.8, 49.4, 116.8, 126.7, 128.6, 129.0, 138.0, 161.3.

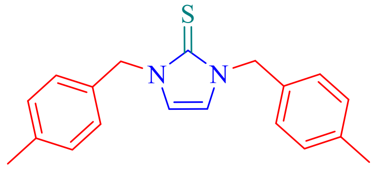

1,3-bis(4-methylbenzyl)-1,3-dihydro-2H-imidazole-2-thione (Table 2, 3s). ^{1}H-NMR (400 MHz, $CDCl_3$) δ (ppm): 2.36 (s, 6H, CH_3), 5.26 (s, 4H, CH_2), 6.52 (s, 2H, CH), 7.18 (d, J=8.0 Hz, 4H, Ar), 7.25 (d, J=8.0 Hz, 4H, Ar). ^{13}C-NMR (100 MHz, $CDCl_3$) δ (ppm): 21.2, 51.2, 116.6, 128.5, 129.6, 132.8, 138.0, 162.9.

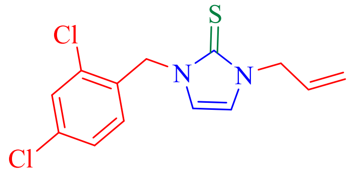

1-allyl-3-(2,4-dichlorobenzyl)-1,3-dihydro-2H-imidazole-2-thione (Table 2, 3t). ^{1}H-NMR (400 MHz, $CDCl_3$) δ (ppm): 4.71 (dt, J_1=6.0, J_2=1.6 Hz, 2H, CH_2), 5.22-5.33 (m, 2H, CH_2), 5.37 (s, 2H, CH_2), 5.90-5.99 (m, 1H, CH), 6.72 (s, 2H, 2CH), 7.22 (dd, J_1=8.4, J_2=2.0 Hz, 1H, Ar), 7.33 (d, J=8.4 Hz, 1H, Ar), 7.42 (d, J=2.4 Hz, 1H, Ar). ^{13}C-NMR (100 MHz, $CDCl_3$) δ (ppm): 48.0, 50.3, 116.8, 116.9, 119.4, 127.7, 129.5, 131.4, 131.7, 132.2, 134.1, 134.7, 163.1.

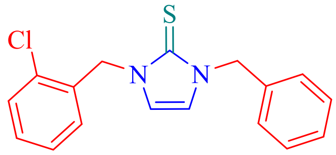

1-benzyl-3-(2-chlorobenzyl)-1,3-dihydro-2H-imidazole-2-thione (Table 2, 3u). ^{1}H-NMR (400 MHz, $CDCl_3$) δ (ppm): 5.32 (s, 2H, CH_2), 5.45 (s, 2H, CH_2), 6.58 (d, J=2.4 Hz, 1H, CH), 6.66 (d, J=2.4 Hz, 1H, CH), 7.25-7.44 (m, 9H, Ar). ^{13}C-NMR (100 MHz, $CDCl_3$) δ (ppm): 48.7, 51.5, 116.8, 117.0, 127.4, 128.2, 128.4, 128.9, 129.6, 129.7, 130.5, 133.5, 133.5, 135.8, 163.5.

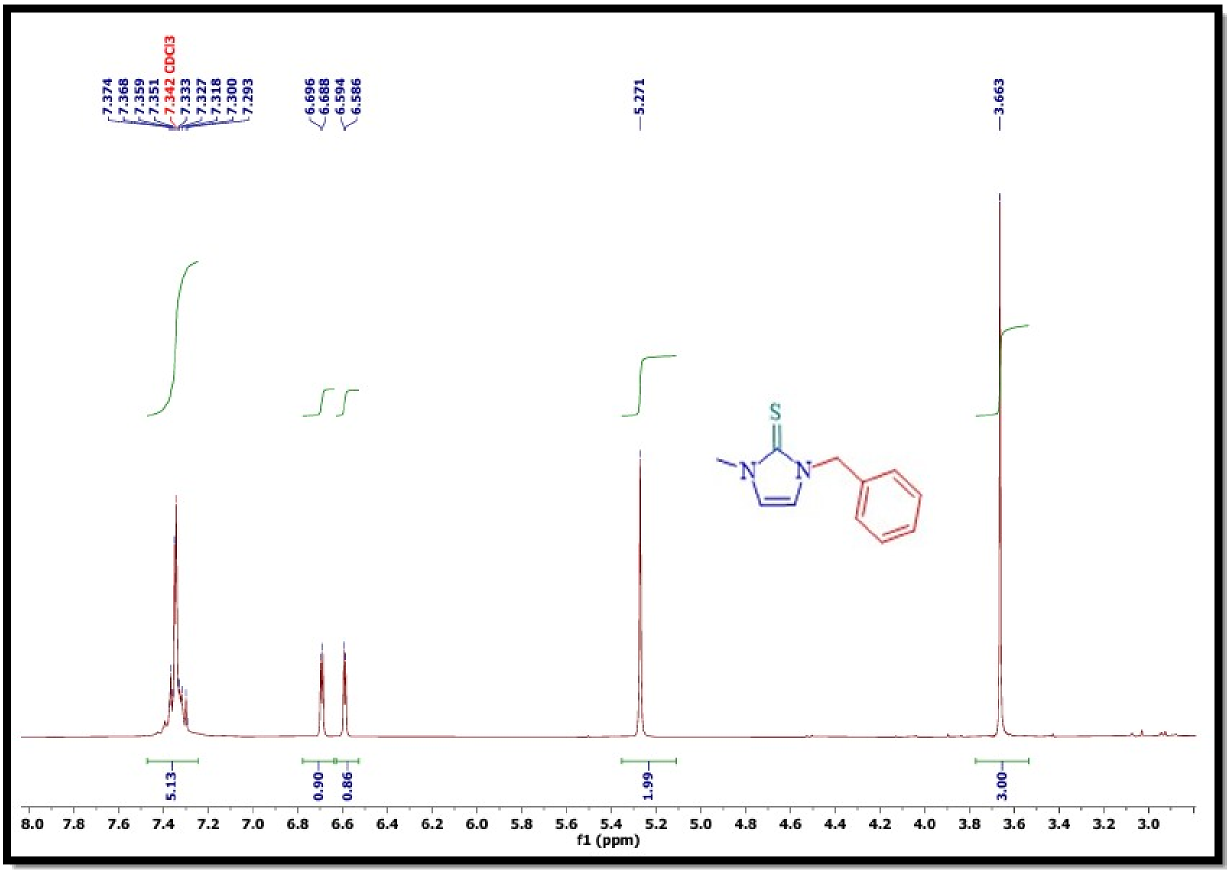

^{1}H-NMR of 1-benzyl-3-methyl-1,3-dihydro-2H-imidazole-2-thione (Table 2, 3a).

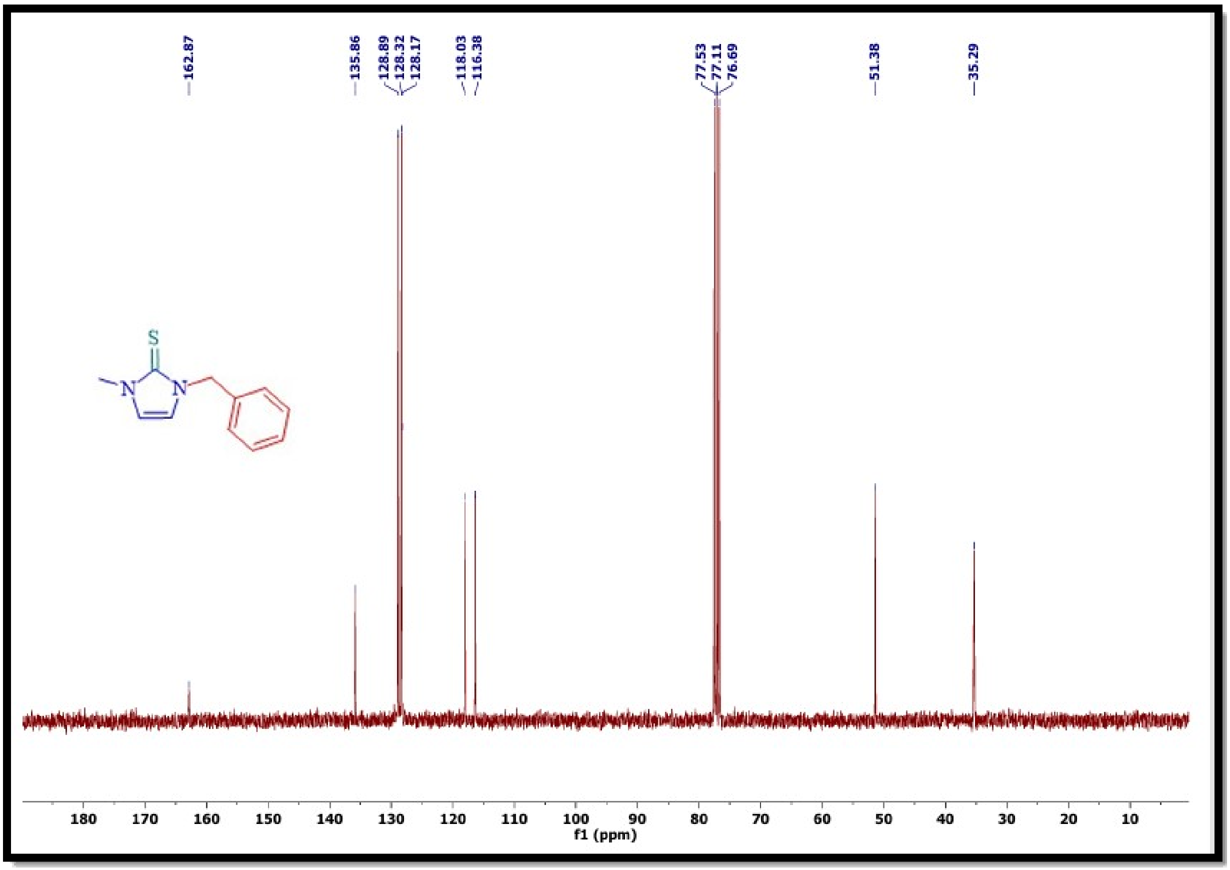

^{13}C-NMR of 1-benzyl-3-methyl-1,3-dihydro-2H-imidazole-2-thione (Table 2, 3a).

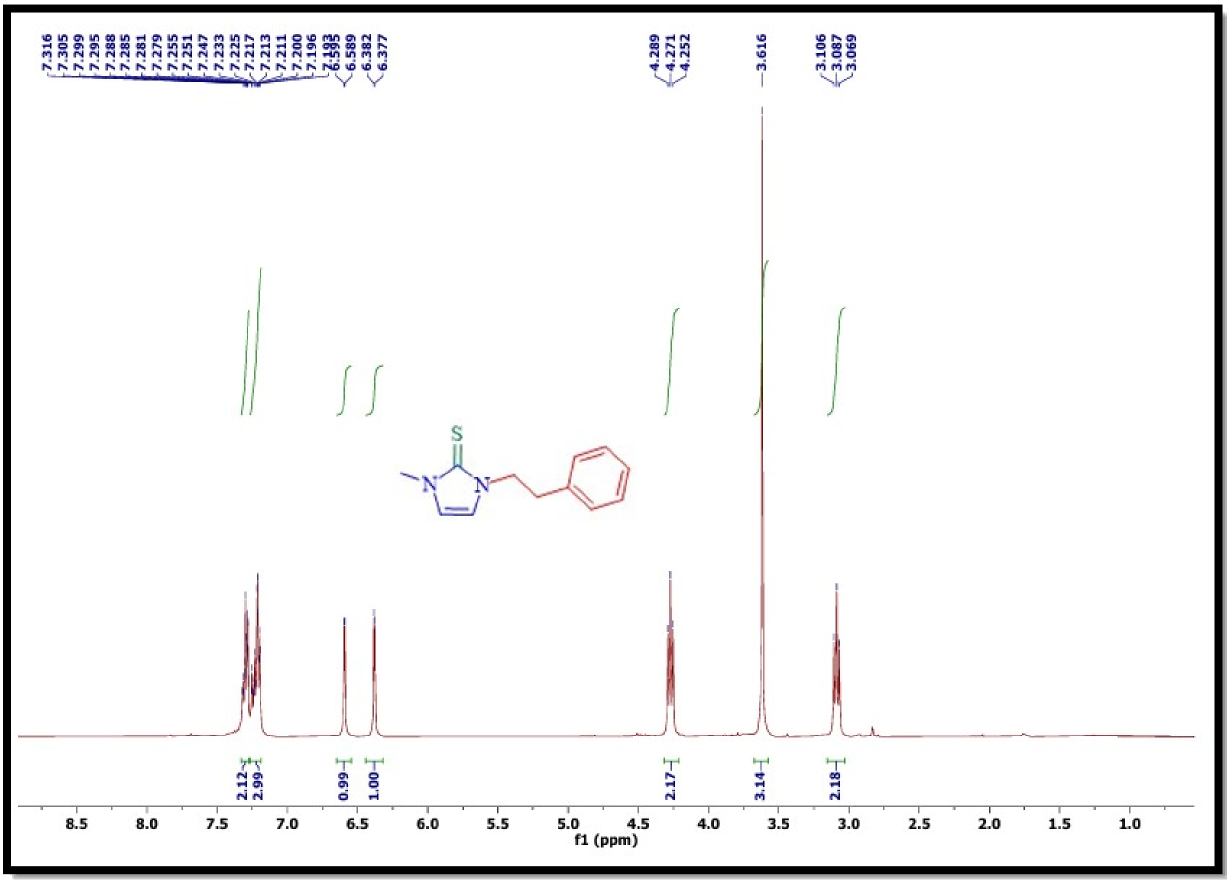

^{1}H-NMR of 1-methyl-3-phenethyl-1,3-dihydro-2H-imidazole-2-thione (Table 2, 3b).

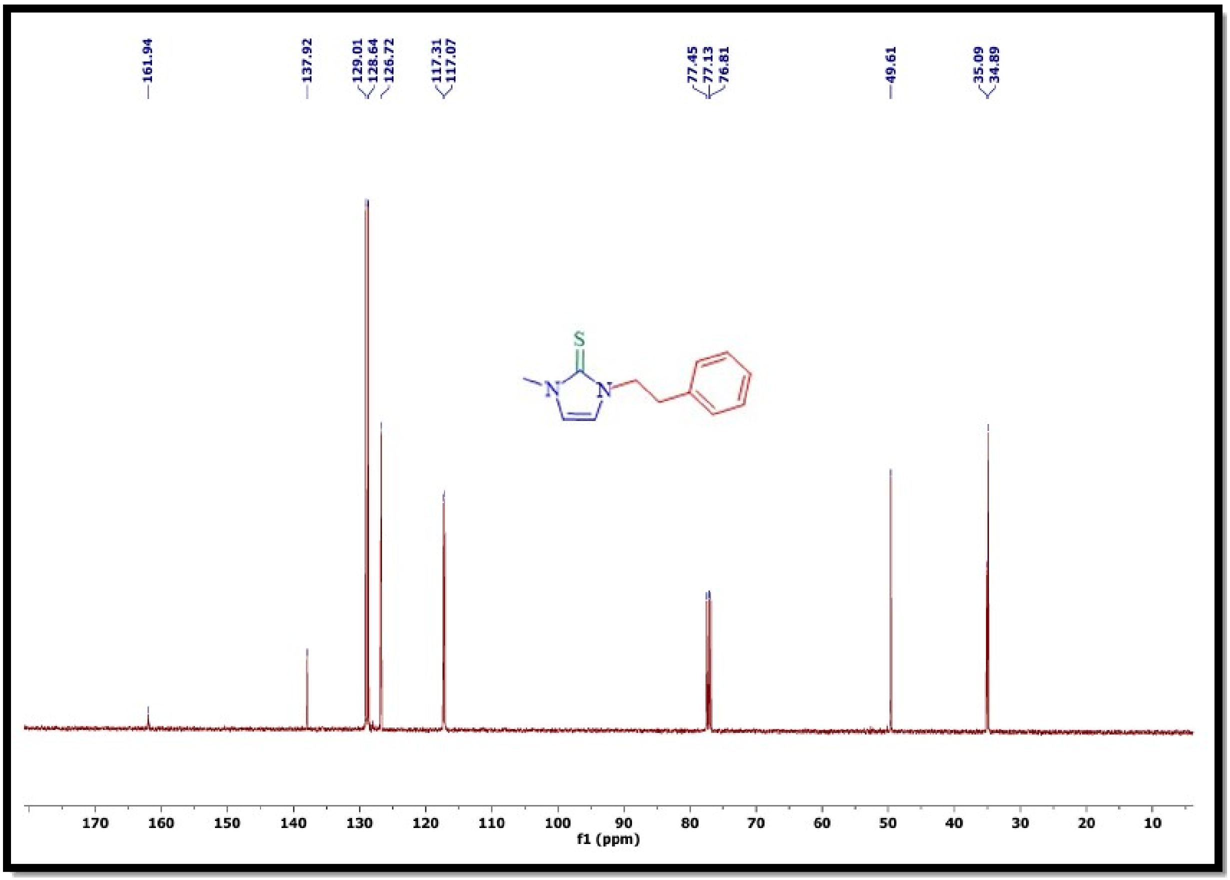

^{13}C-NMR of 1-methyl-3-phenethyl-1,3-dihydro-2H-imidazole-2-thione (Table 2, 3b).

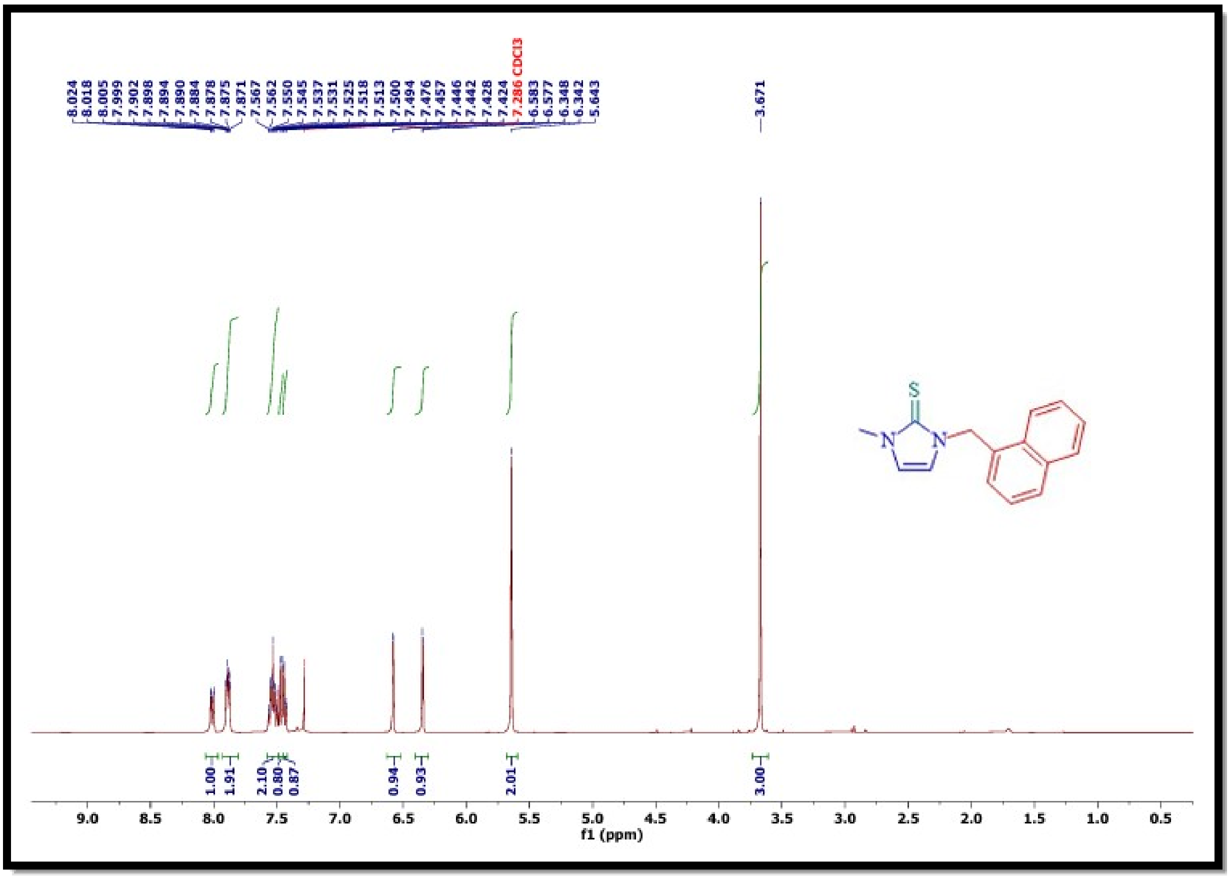

^{1}H-NMR of 1-methyl-3-(naphthalen-1-ylmethyl)-1,3-dihydro-2H-imidazole-2-thione (Table 2, 3c).

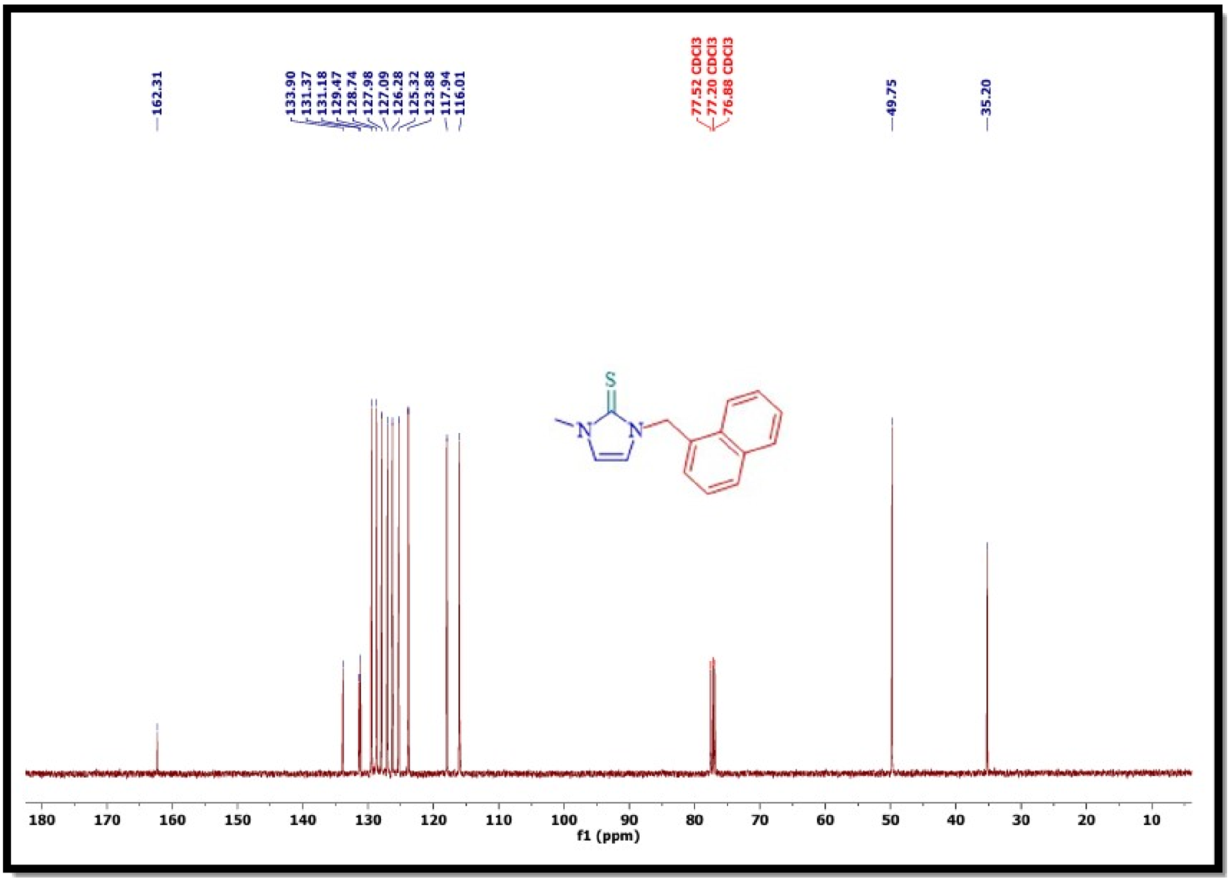

^{13}C-NMR of 1-methyl-3-(naphthalen-1-ylmethyl)-1,3-dihydro-2H-imidazole-2-thione (Table 2, 3c).

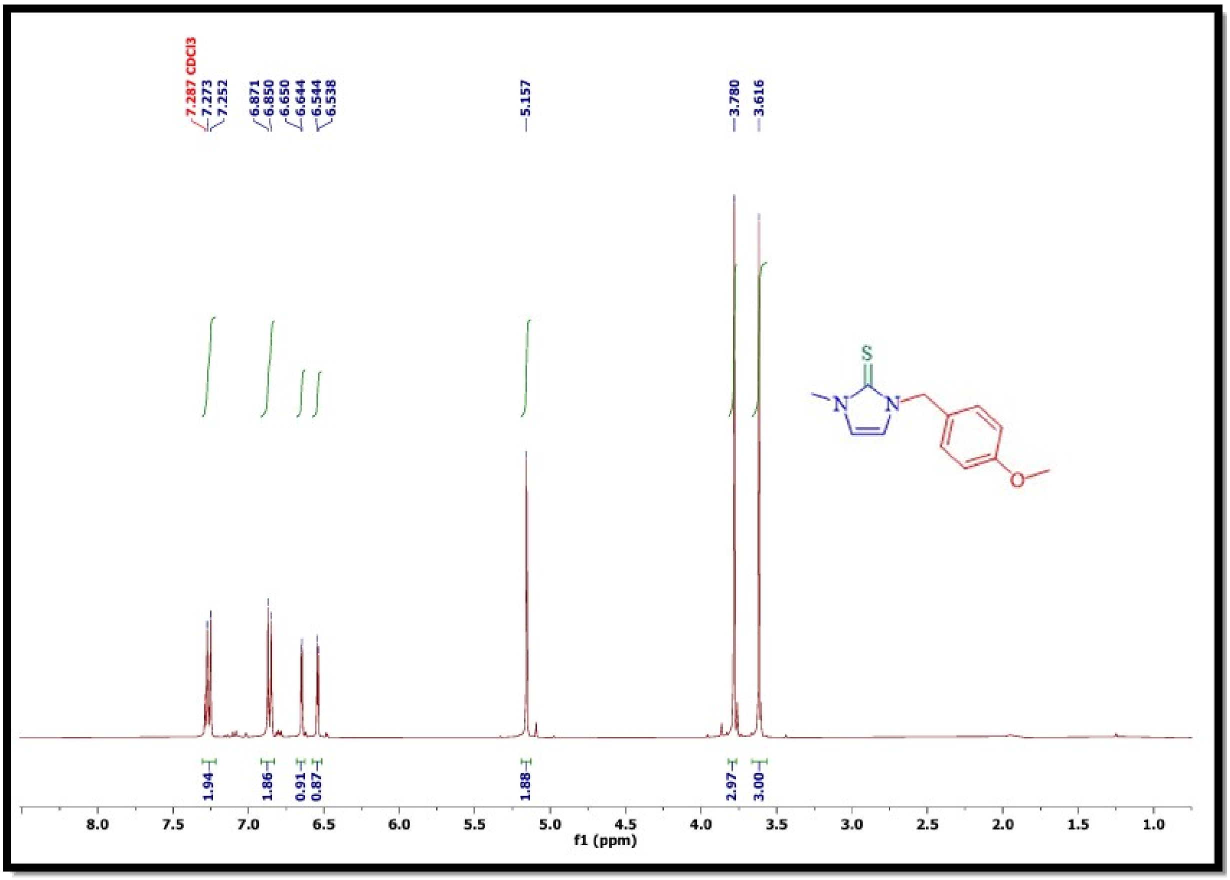

^{1}H-NMR of 1-(4-methoxybenzyl)-3-methyl-1,3-dihydro-2H-imidazole-2-thione (Table 2, 3d).

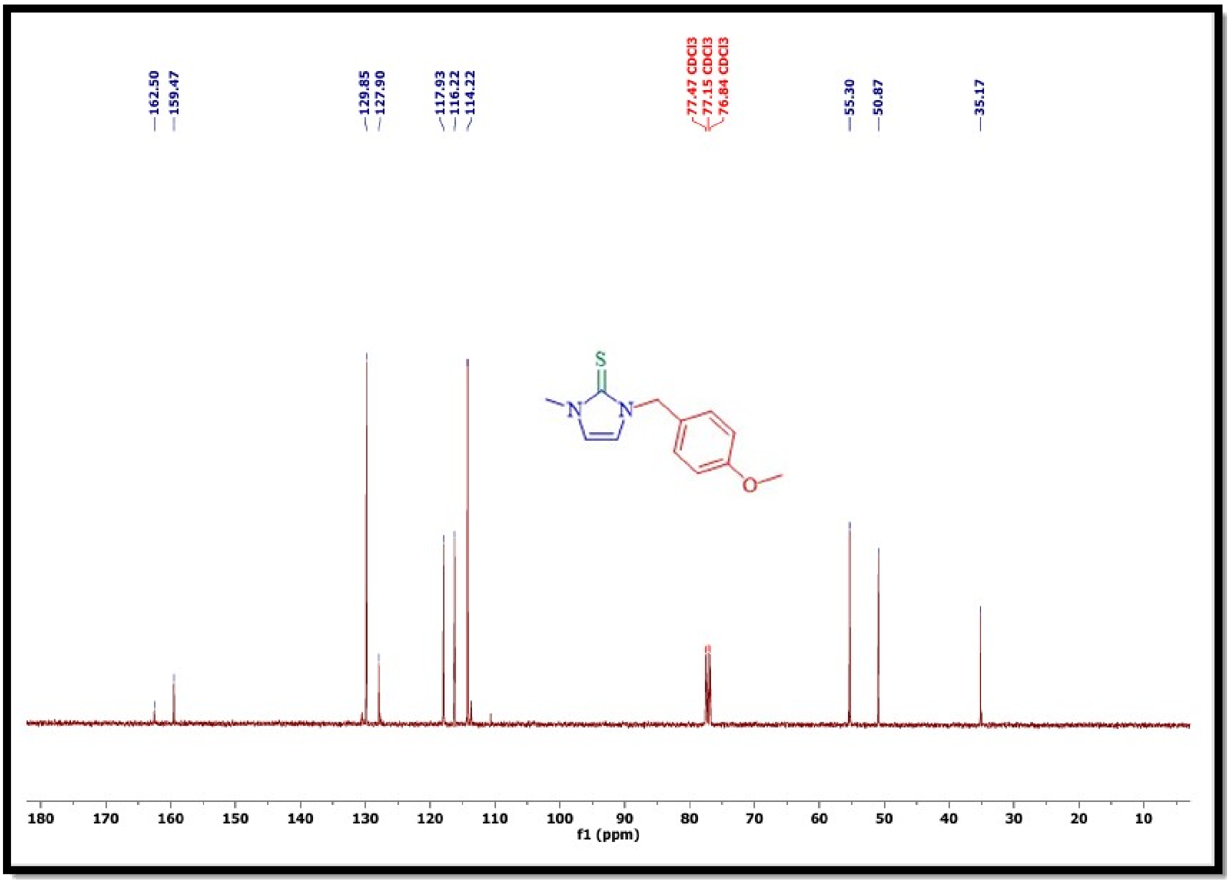

^{13}C-NMR of 1-(4-methoxybenzyl)-3-methyl-1,3-dihydro-2H-imidazole-2-thione (Table 2, 3d).

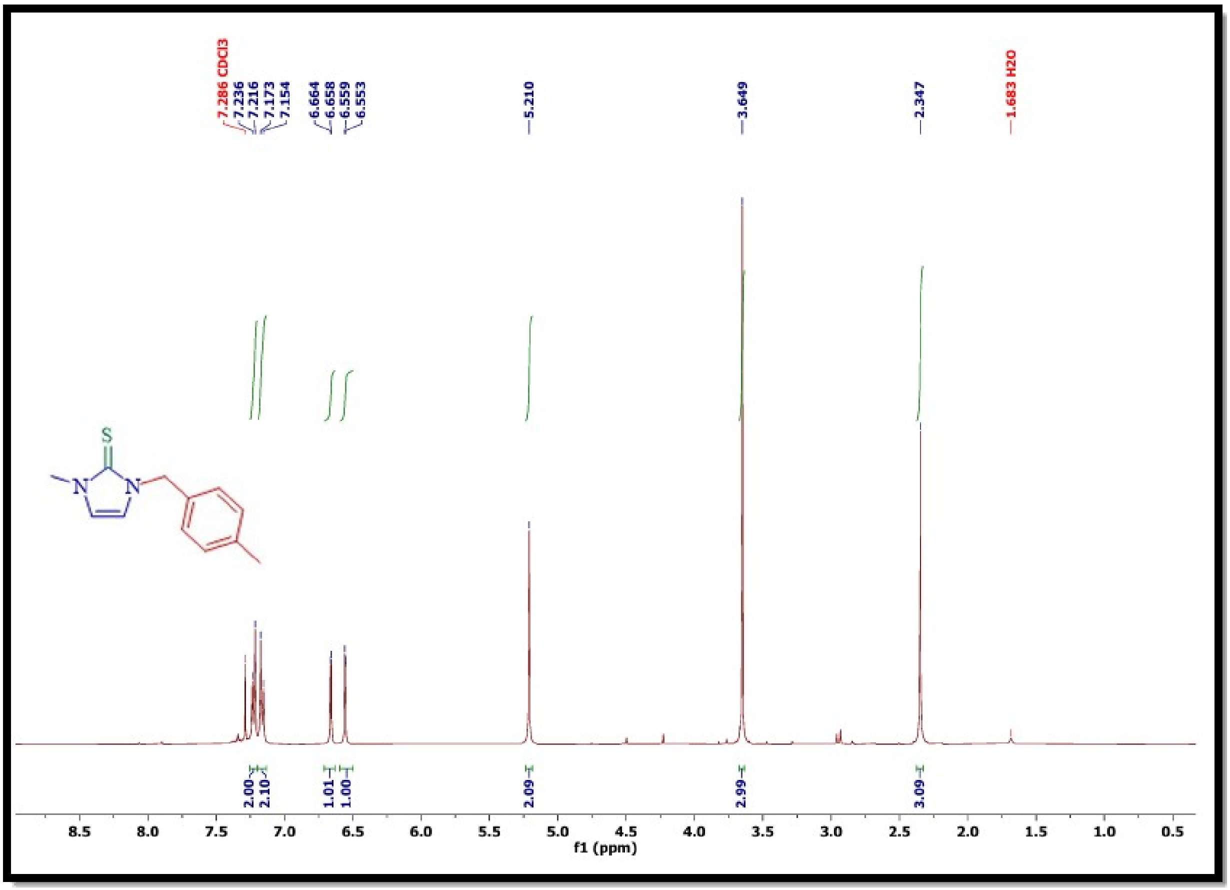

^{1}H-NMR of 1-methyl-3-(4-methylbenzyl)-1,3-dihydro-2H-imidazole-2-thione (Table 2, 3e).

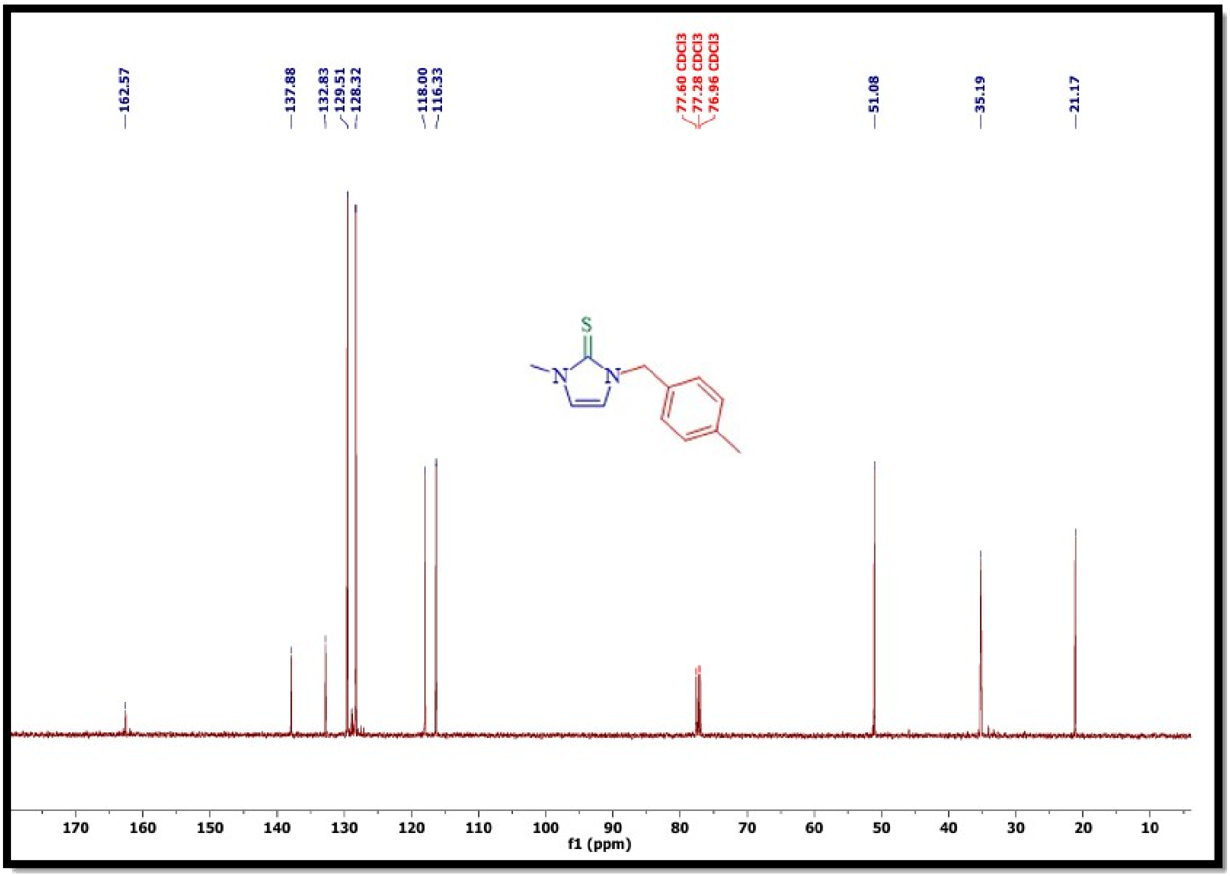

^{13}C-NMR of 1-methyl-3-(4-methylbenzyl)-1,3-dihydro-2H-imidazole-2-thione (Table 2, 3e).

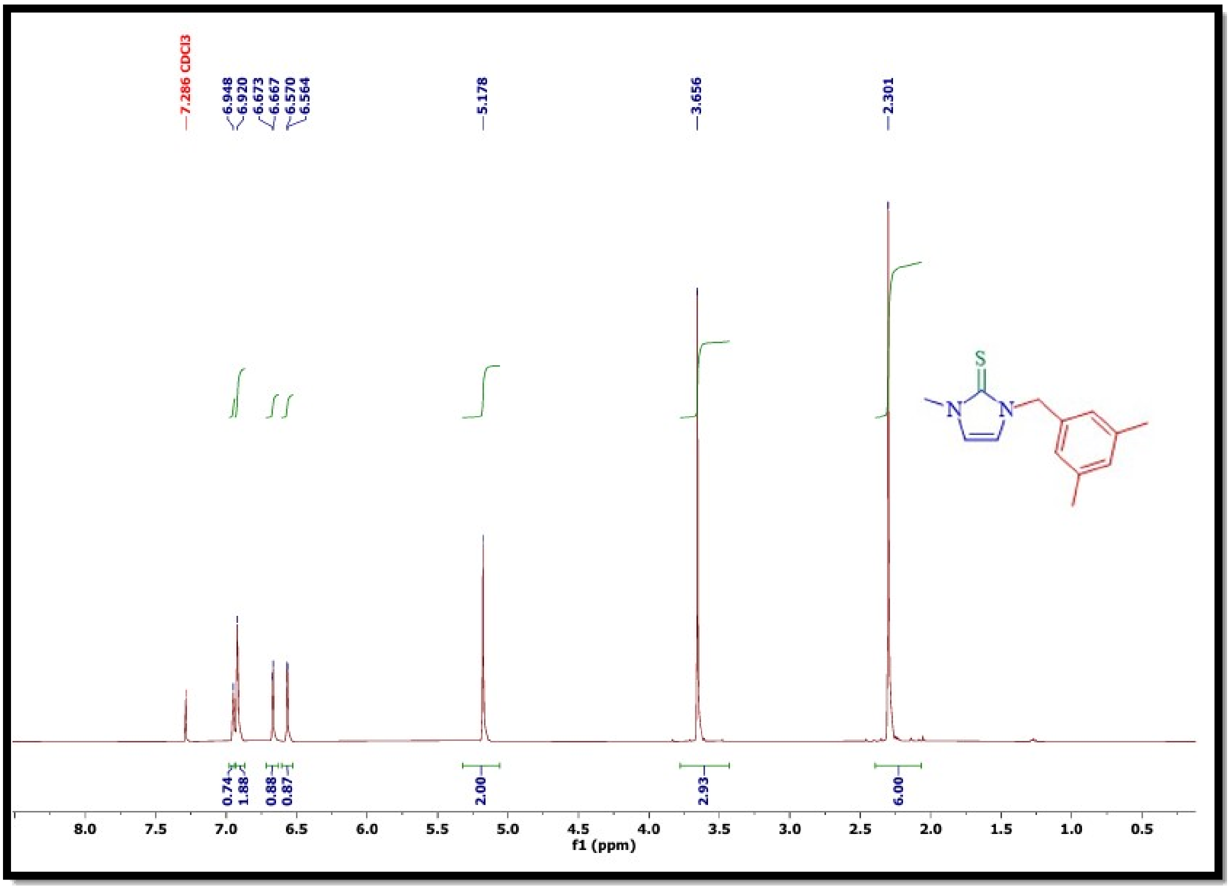

^{1}H-NMR of 1-(3,5-dimethylbenzyl)-3-methyl-1,3-dihydro-2H-imidazole-2-thione (Table 2, 3f).

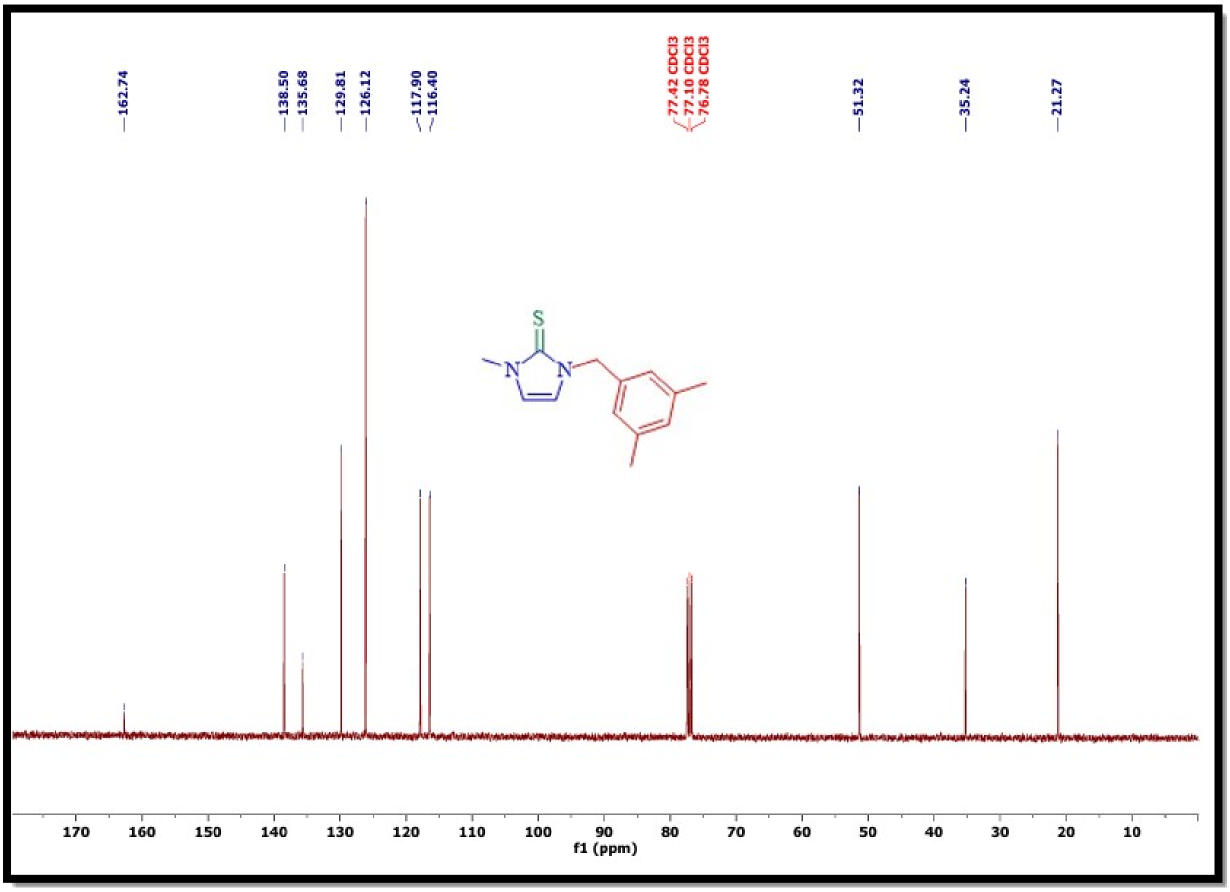

^{13}C-NMR of 1-(3,5-dimethylbenzyl)-3-methyl-1,3-dihydro-2H-imidazole-2-thione (Table 2, 3f).

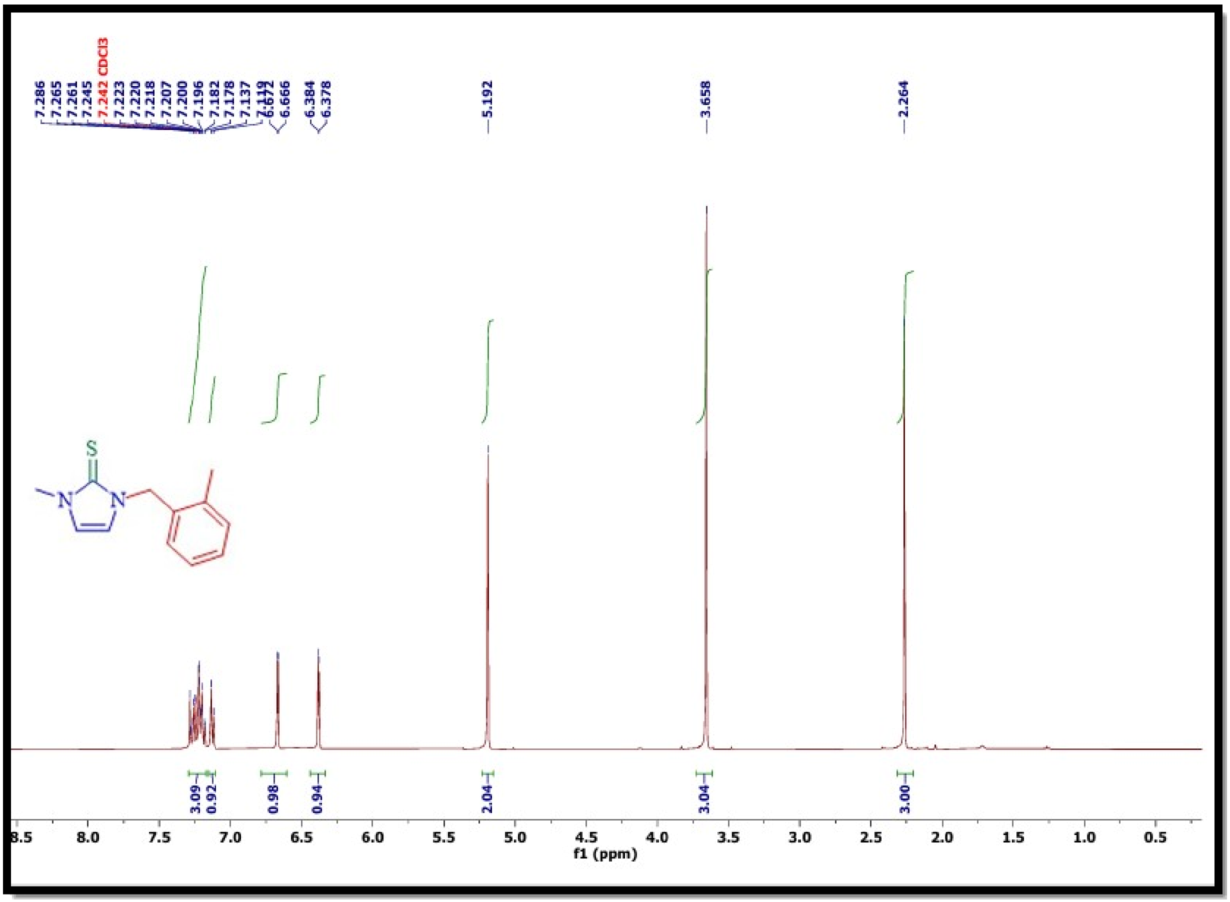

^{1}H-NMR of 1-methyl-3-(2-methylbenzyl)-1,3-dihydro-2H-imidazole-2-thione (Table 2, 3g).

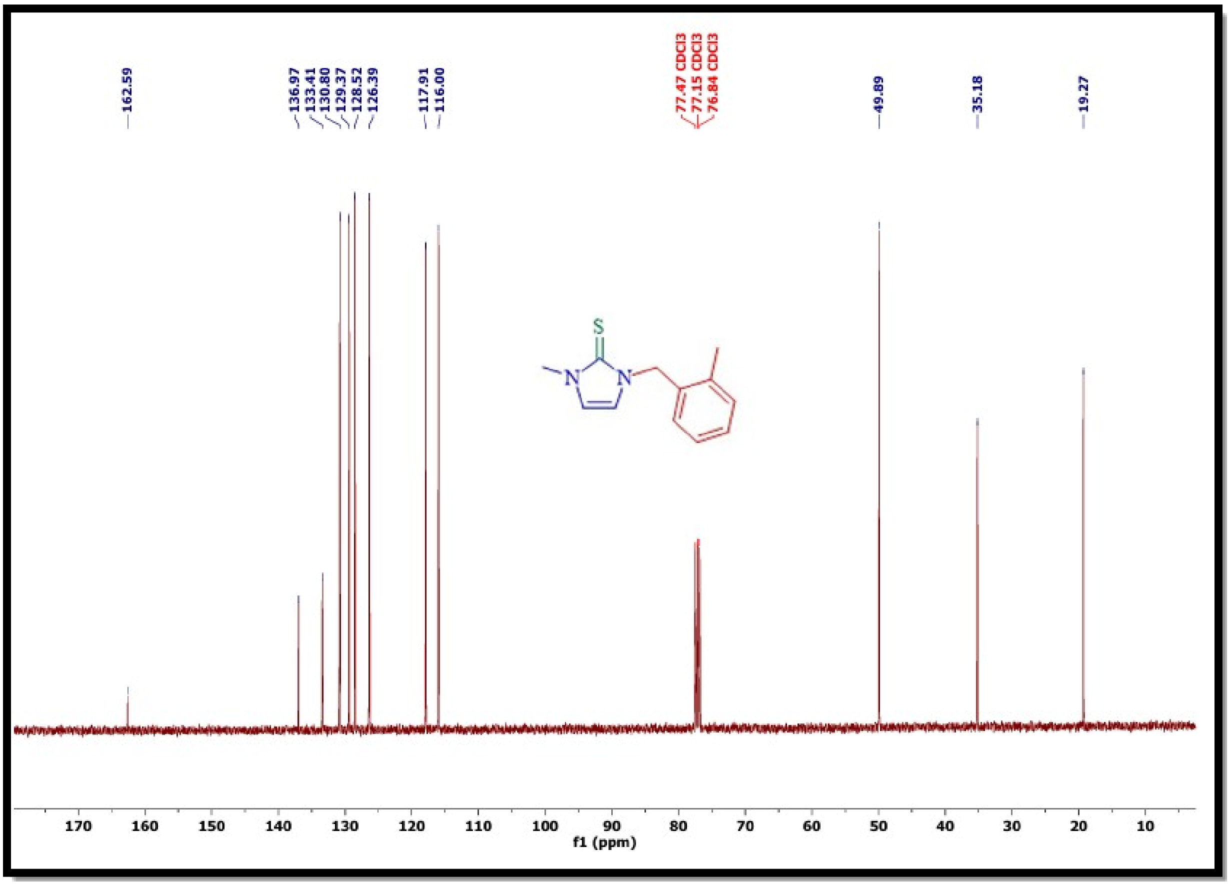

^{13}C-NMR of 1-methyl-3-(2-methylbenzyl)-1,3-dihydro-2H-imidazole-2-thione (Table 2, 3g).

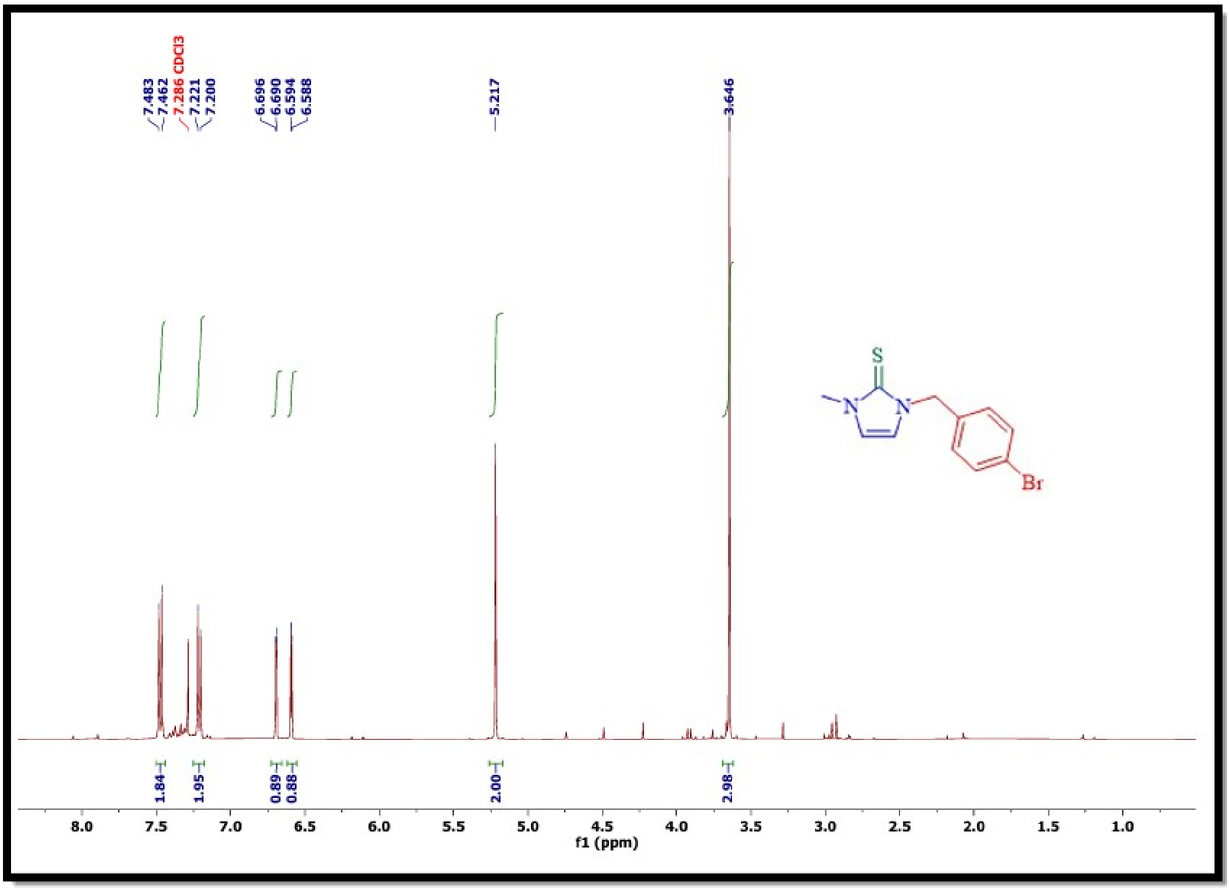

^{1}H-NMR of 1-(4-bromobenzyl)-3-methyl-1,3-dihydro-2H-imidazole-2-thione (Table 2, 3h).

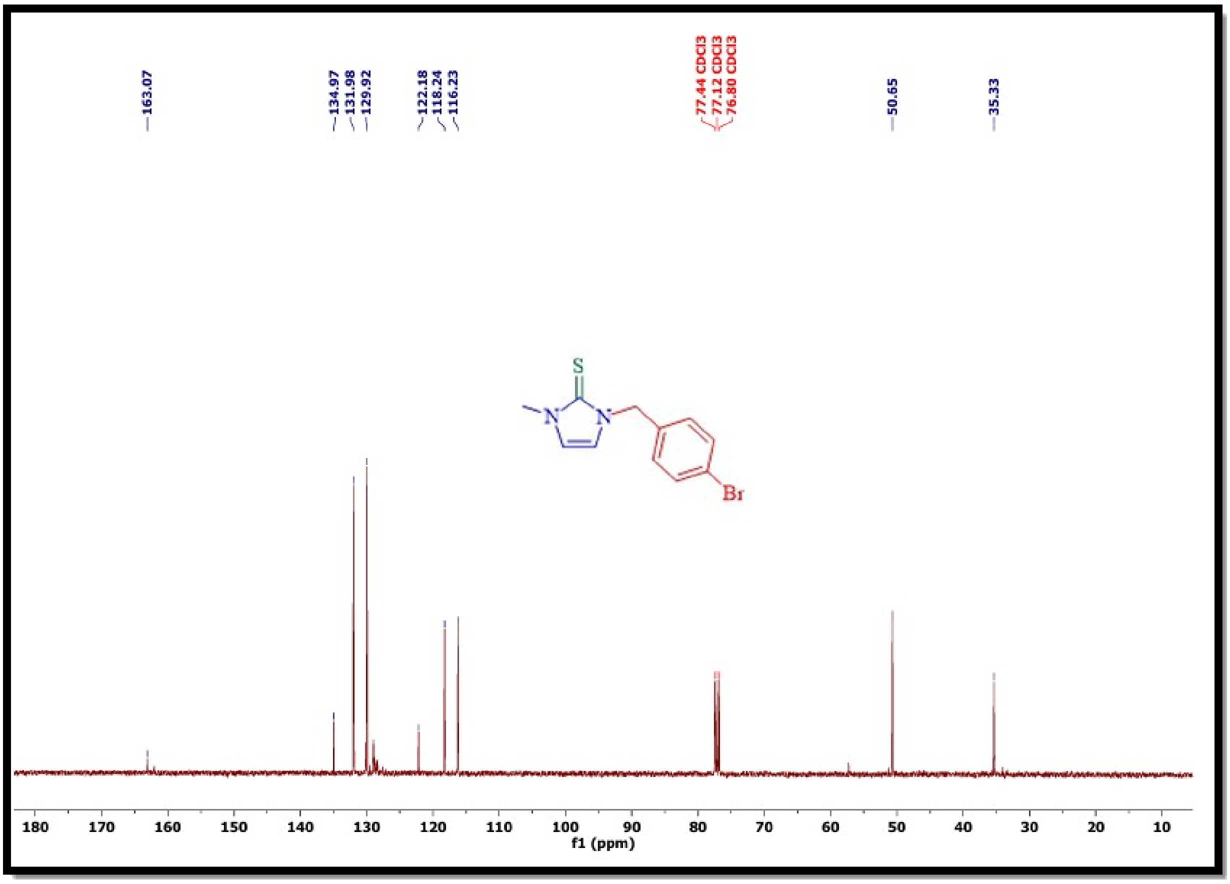

^{13}C-NMR of 1-(4-bromobenzyl)-3-methyl-1,3-dihydro-2H-imidazole-2-thione (Table 2, 3h).

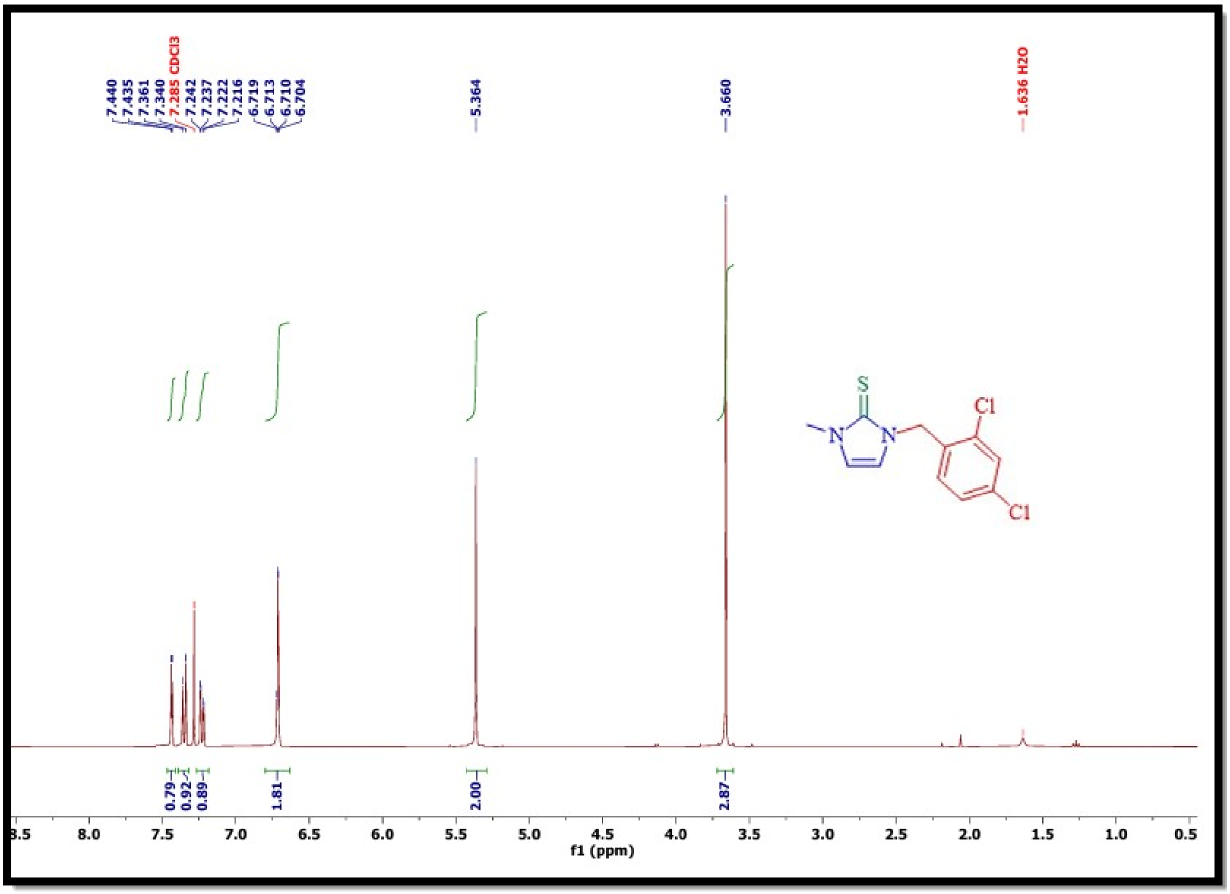

^{1}H-NMR of 1-(2,4-dichlorobenzyl)-3-methyl-1,3-dihydro-2H-imidazole-2-thione (Table 2, 3i).

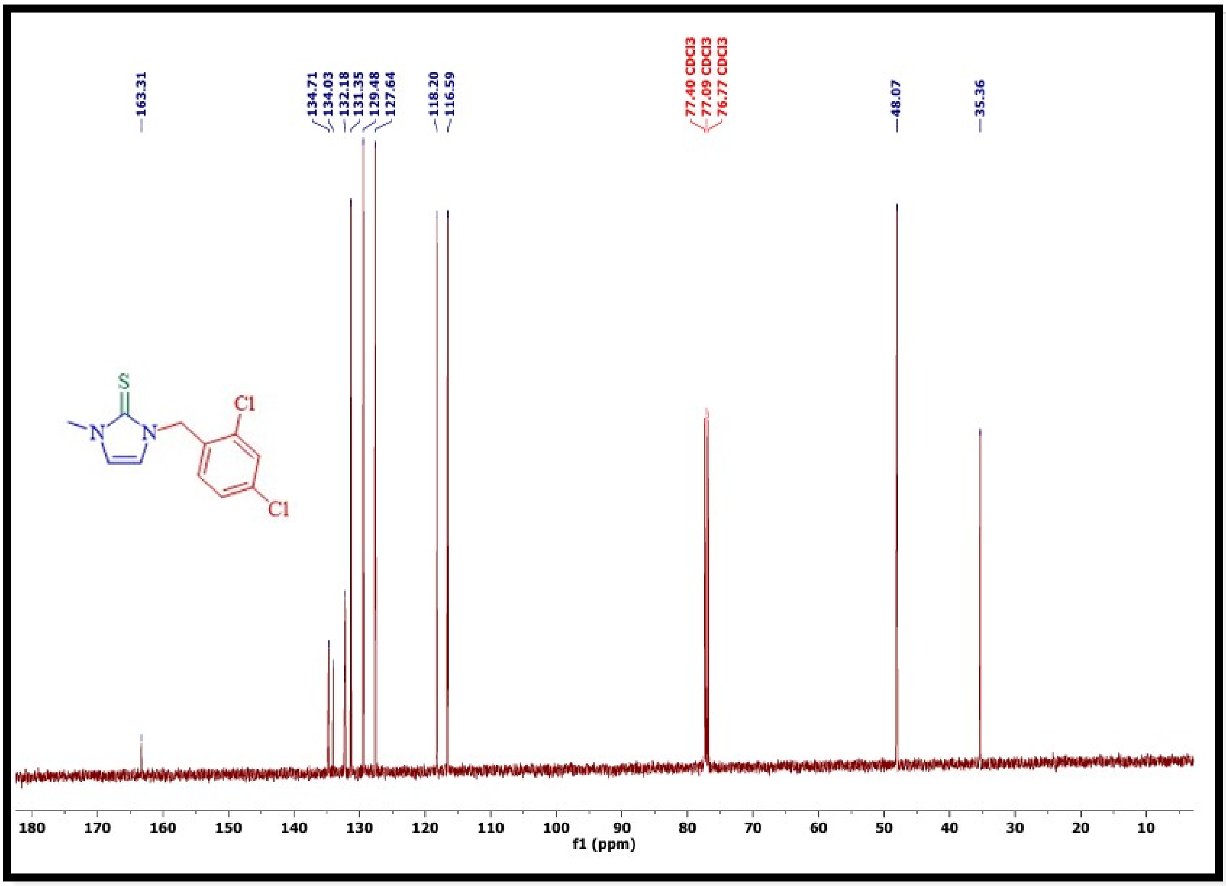

^{13}C-NMR of 1-(2,4-dichlorobenzyl)-3-methyl-1,3-dihydro-2H-imidazole-2-thione (Table 2, 3i).

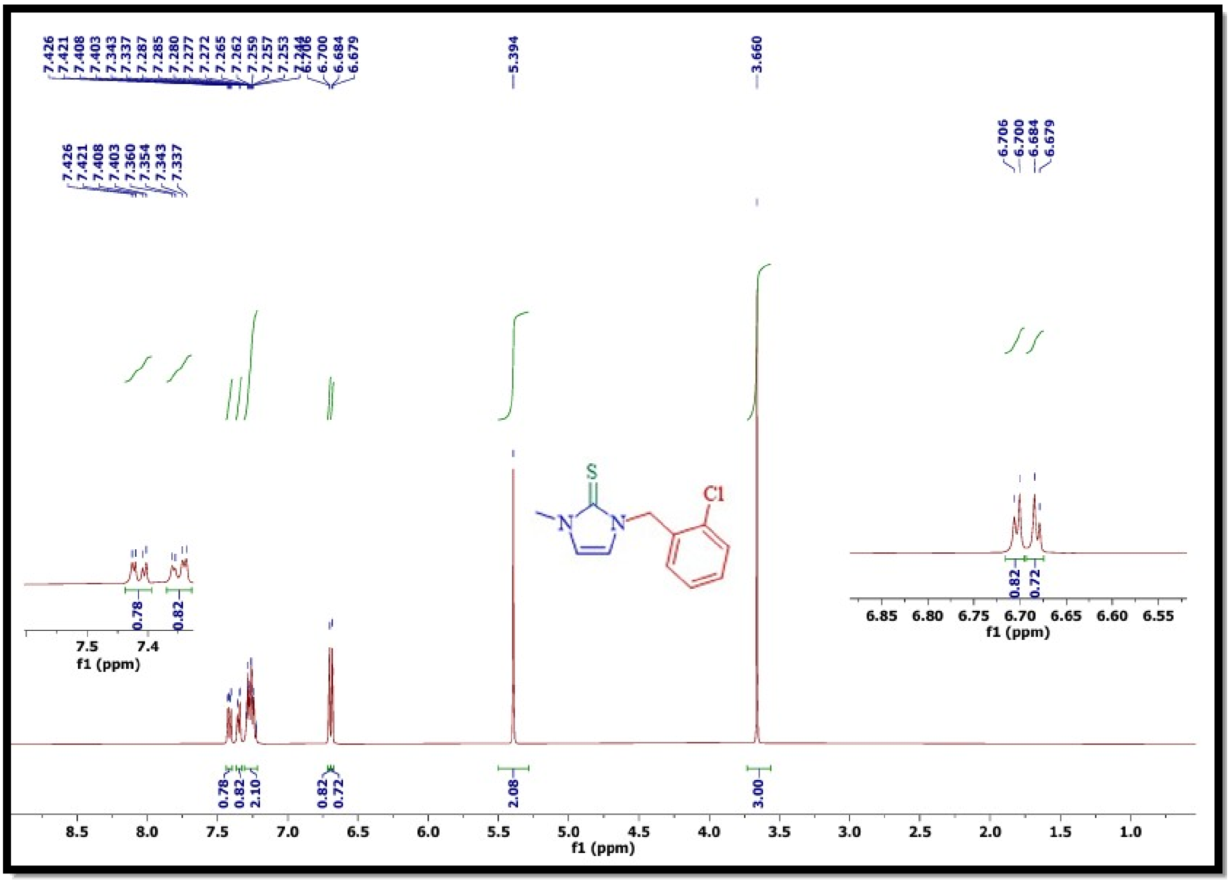

^{1}H-NMR of 1-(2-chlorobenzyl)-3-methyl-1,3-dihydro-2H-imidazole-2-thione (Table 2, 3j).

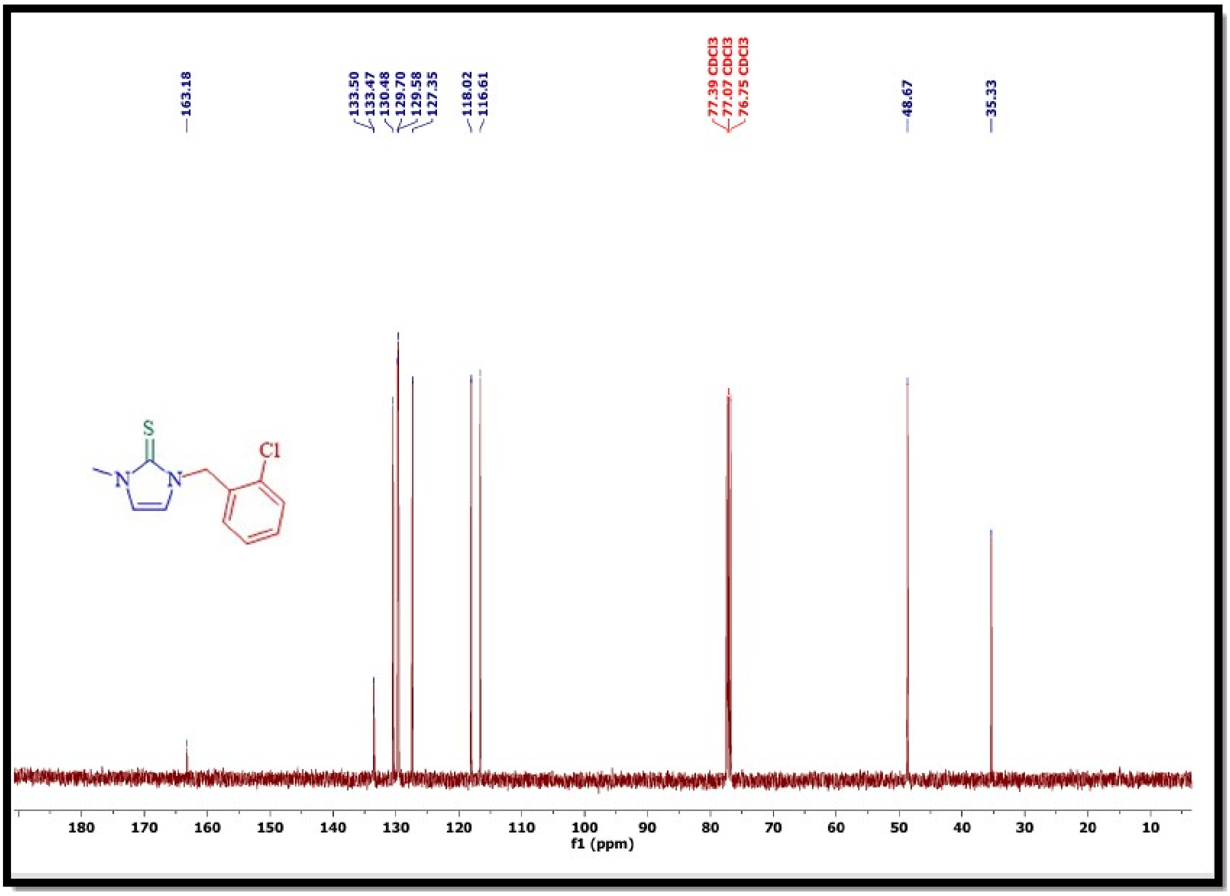

^{13}C-NMR of 1-(2-chlorobenzyl)-3-methyl-1,3-dihydro-2H-imidazole-2-thione (Table 2, 3j).

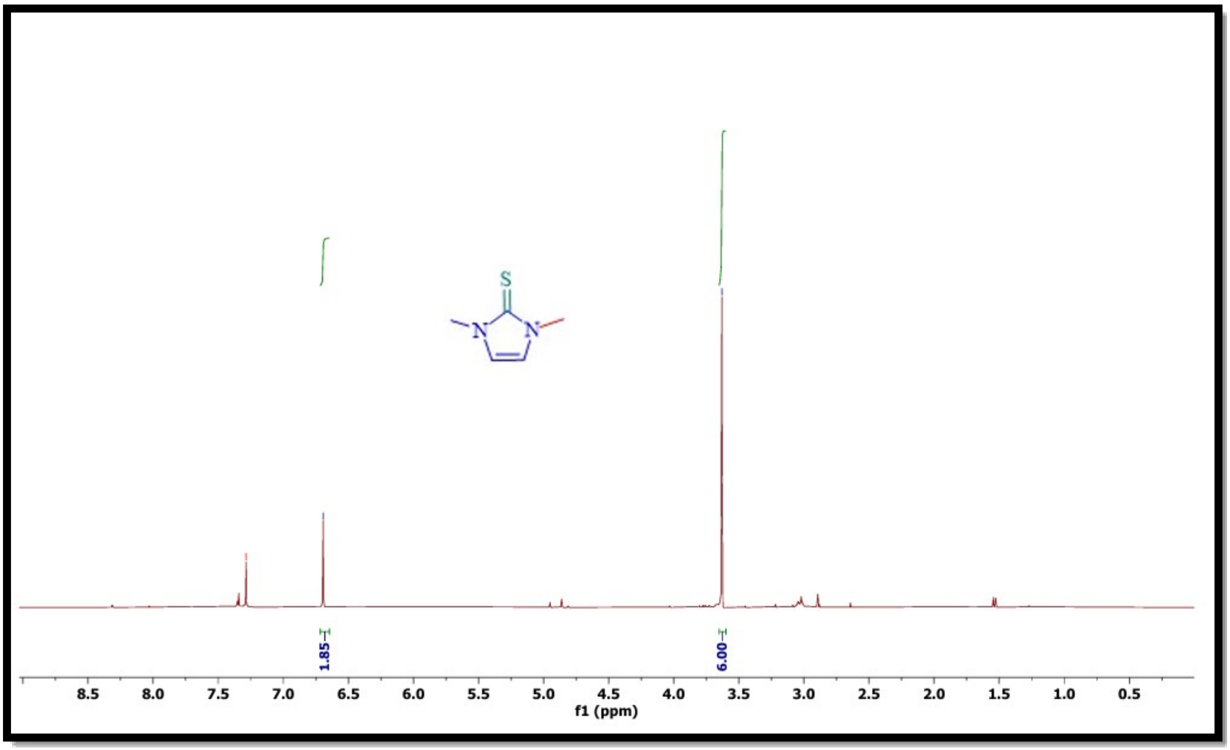

^{1}H-NMR of 1,3-dimethyl-1,3-dihydro-2H-imidazole-2-thione (Table 2, 3l).

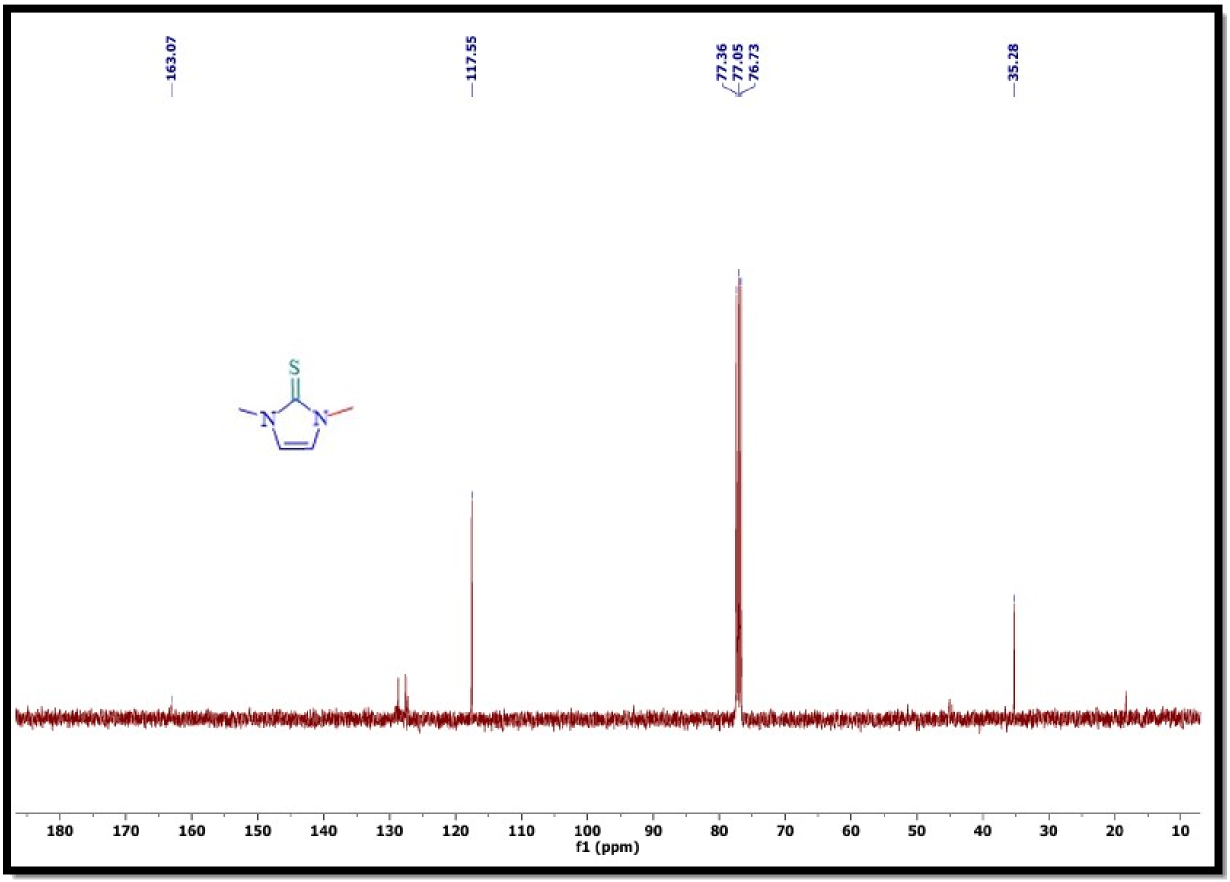

^{13}C-NMR of 1,3-dimethyl-1,3-dihydro-2H-imidazole-2-thione (Table 2, 3l).

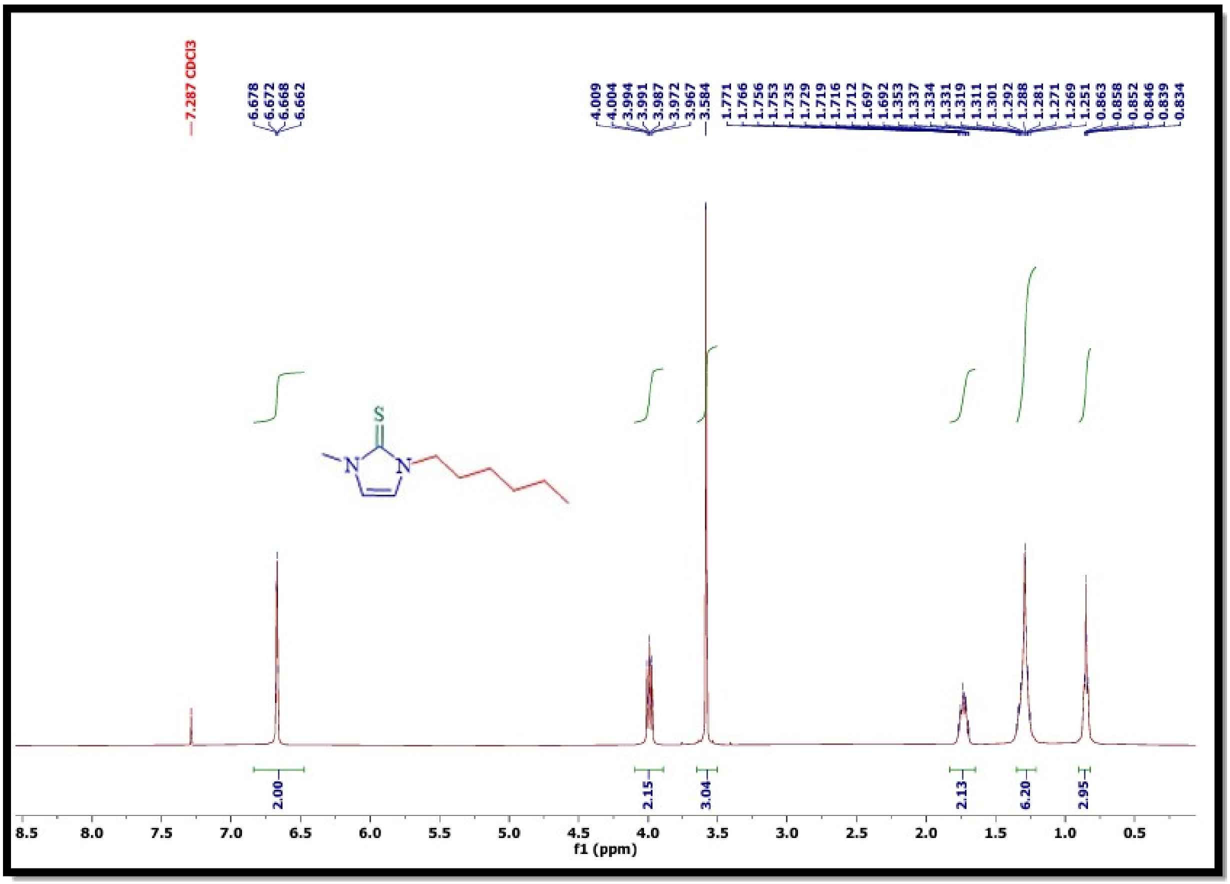

^{1}H-NMR of 1-hexyl-3-methyl-1,3-dihydro-2H-imidazole-2-thione (Table 2, 3m).

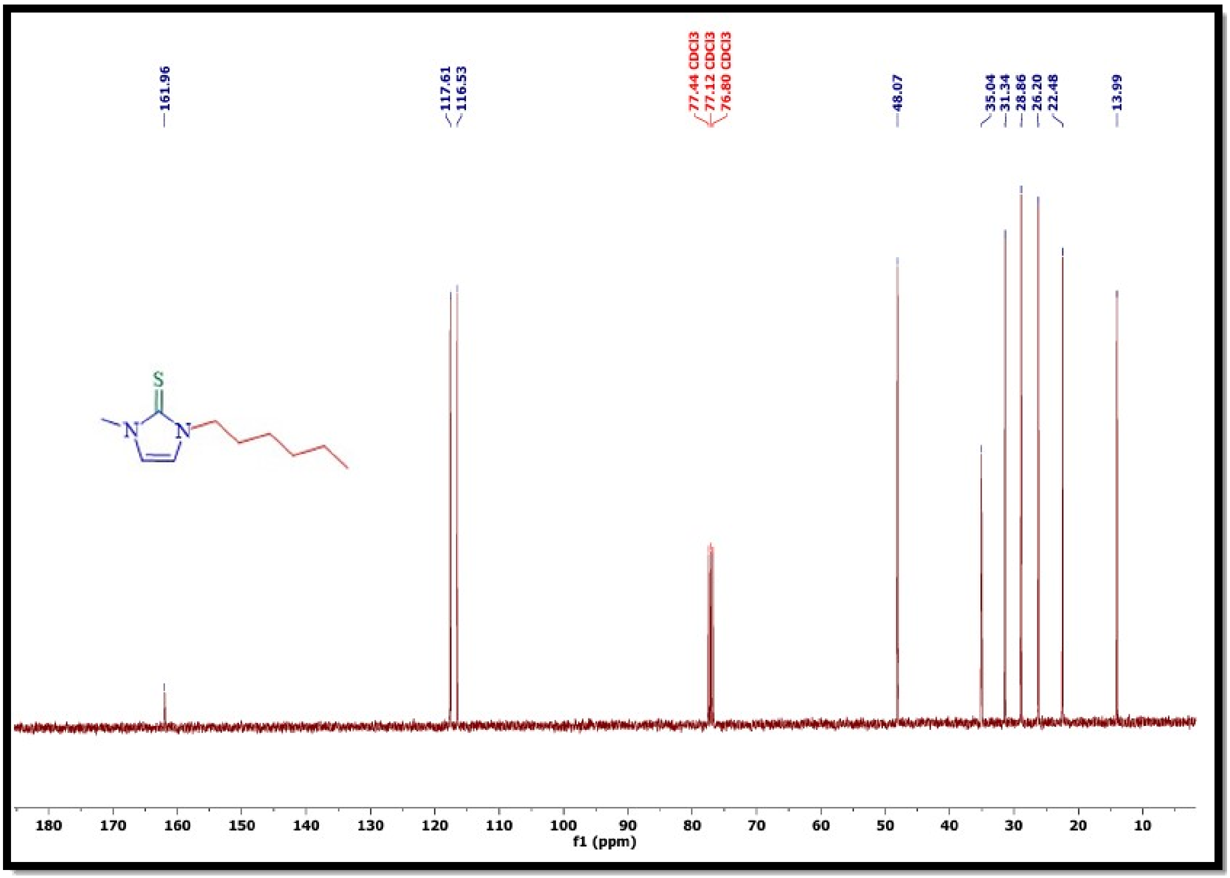

^{13}C-NMR of 1-hexyl-3-methyl-1,3-dihydro-2H-imidazole-2-thione (Table 2, 3m).

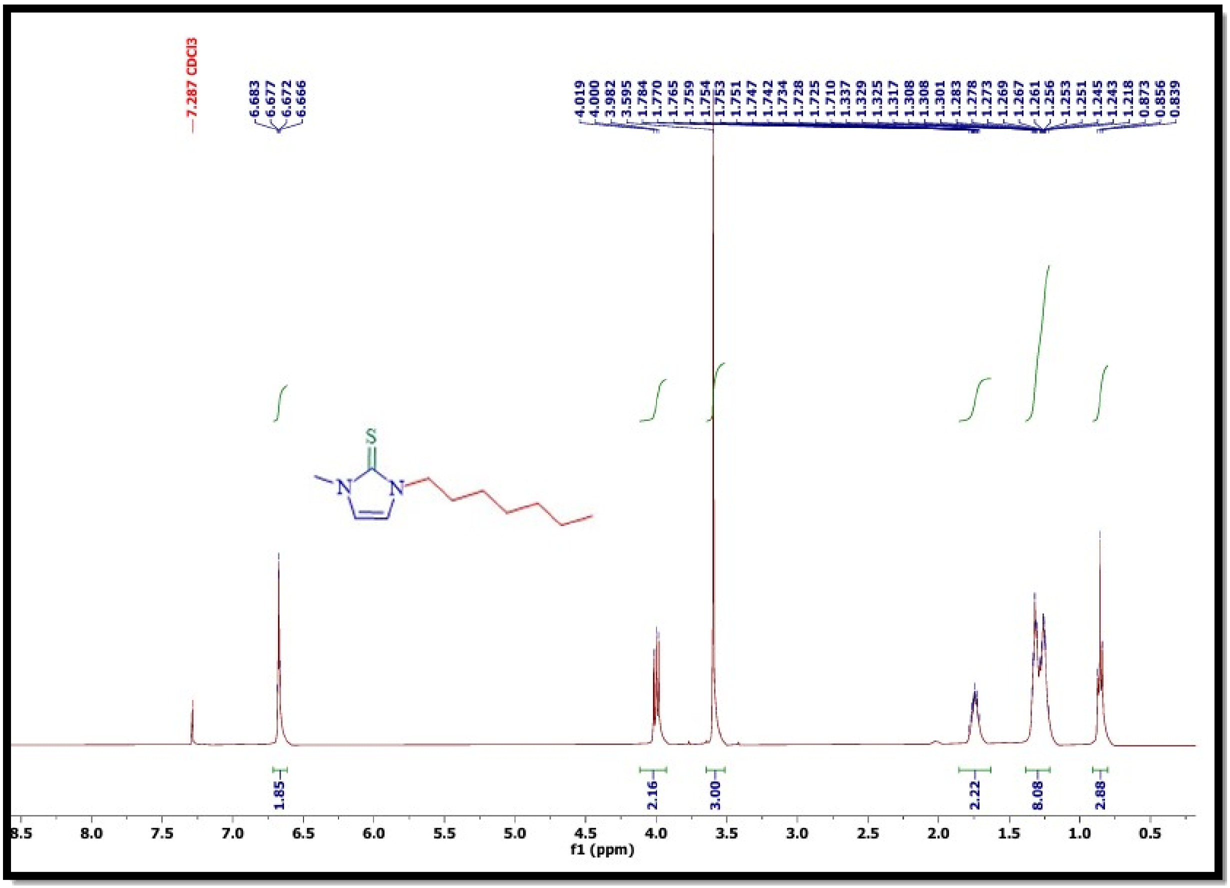

^{1}H-NMR of 1-heptyl-3-methyl-1,3-dihydro-2H-imidazole-2-thione (Table 2, 3n).

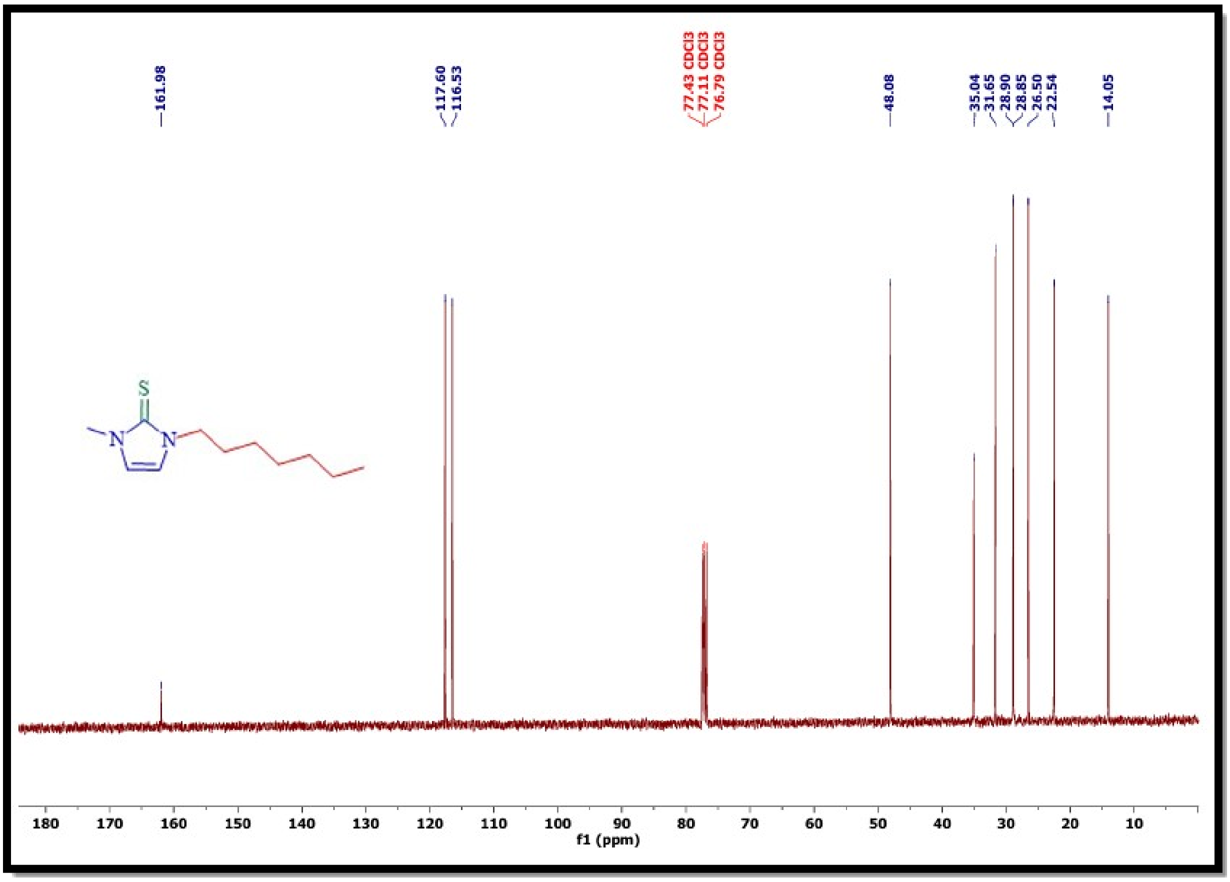

^{13}C-NMR of 1-heptyl-3-methyl-1,3-dihydro-2H-imidazole-2-thione (Table 2, 3n).

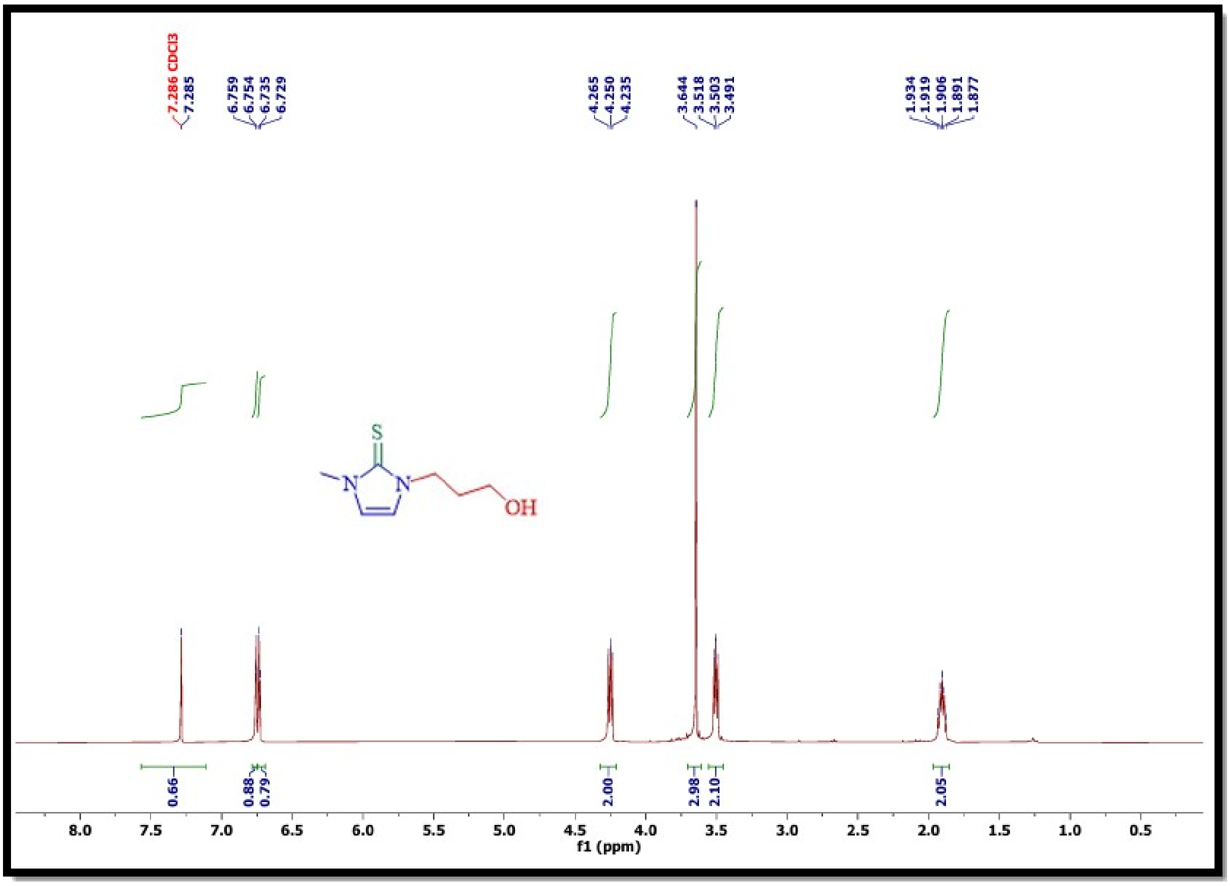

^{1}H-NMR of 1-(3-hydroxypropyl)-3-methyl-1,3-dihydro-2H-imidazole-2-thione (Table 2, 3o).

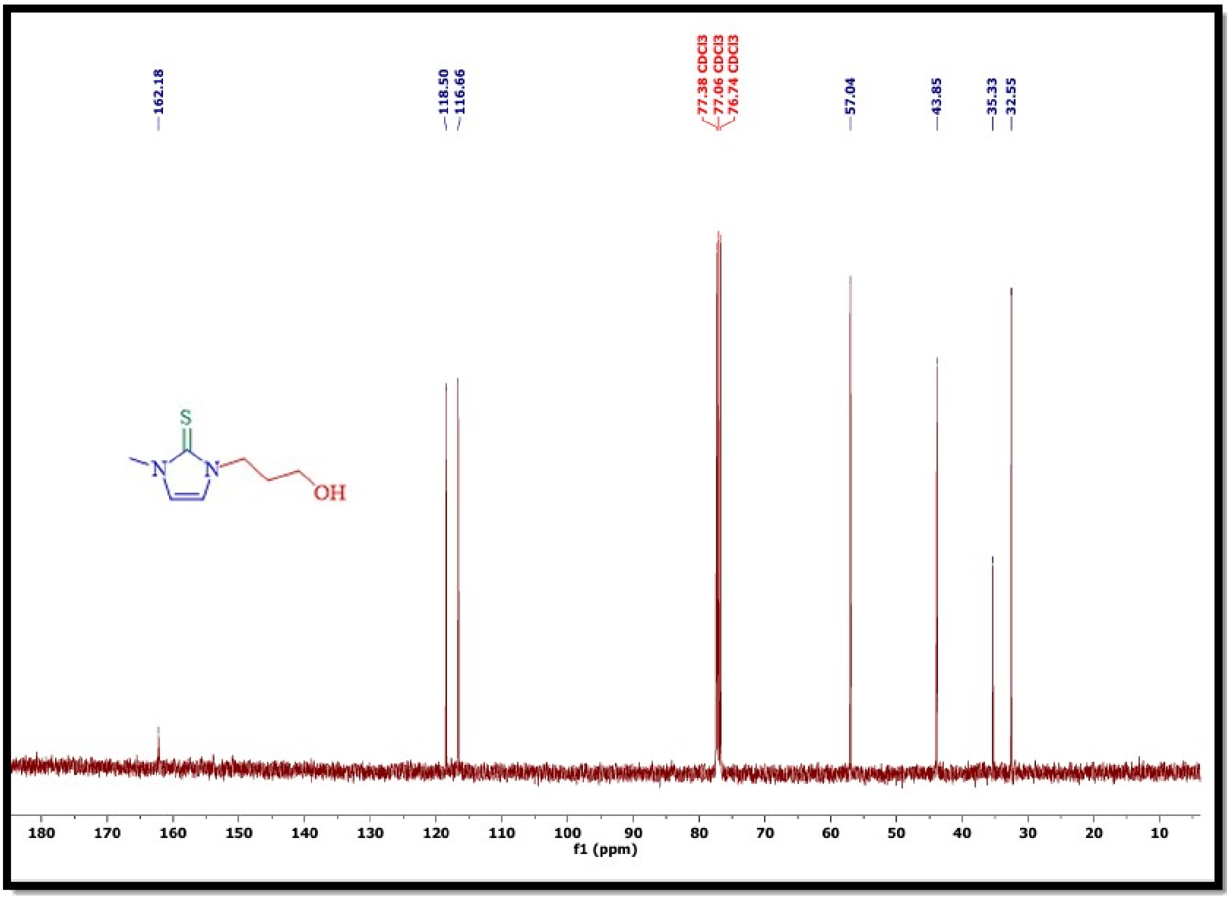

^{13}C-NMR of 1-(3-hydroxypropyl)-3-methyl-1,3-dihydro-2H-imidazole-2-thione (Table 2, 3o).

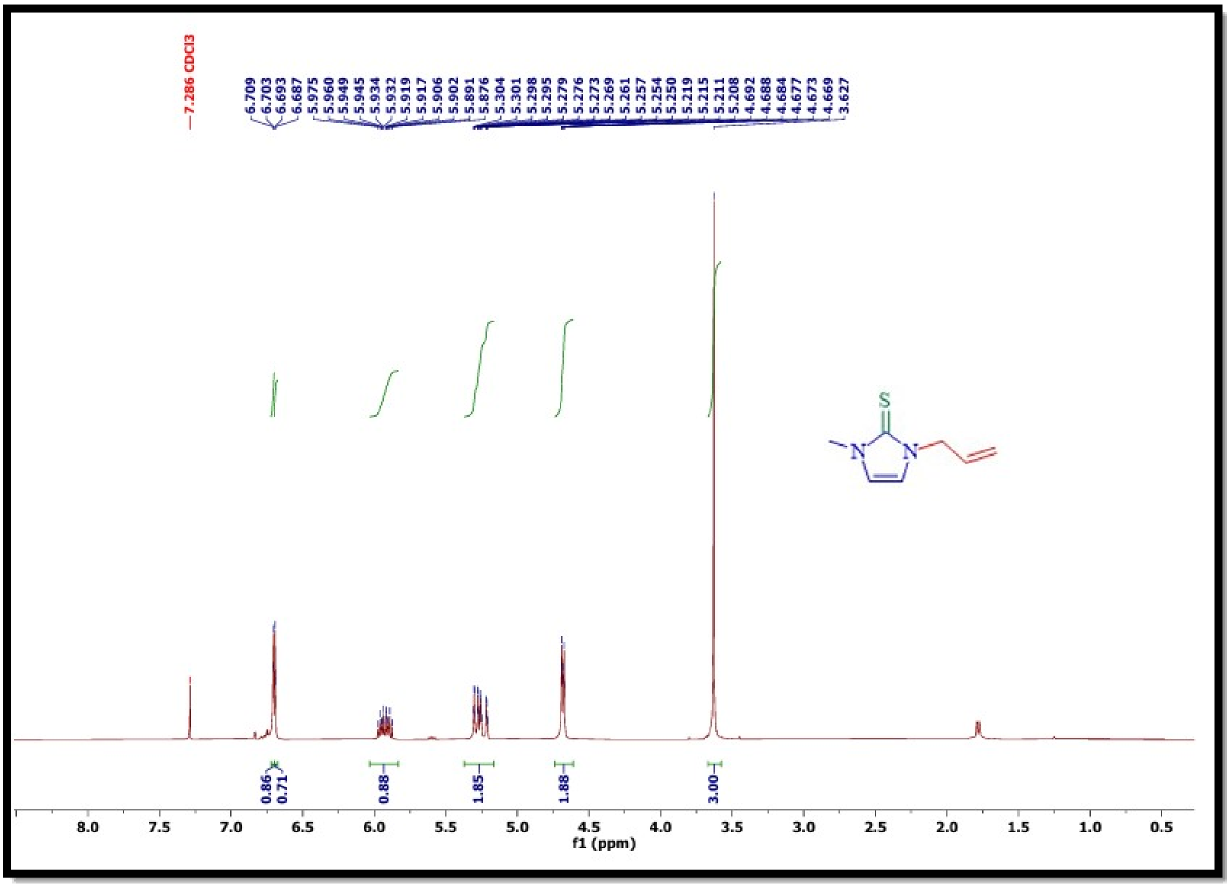

^{1}H-NMR of 1-allyl-3-methyl-1,3-dihydro-2H-imidazole-2-thione (Table 2, 3p).

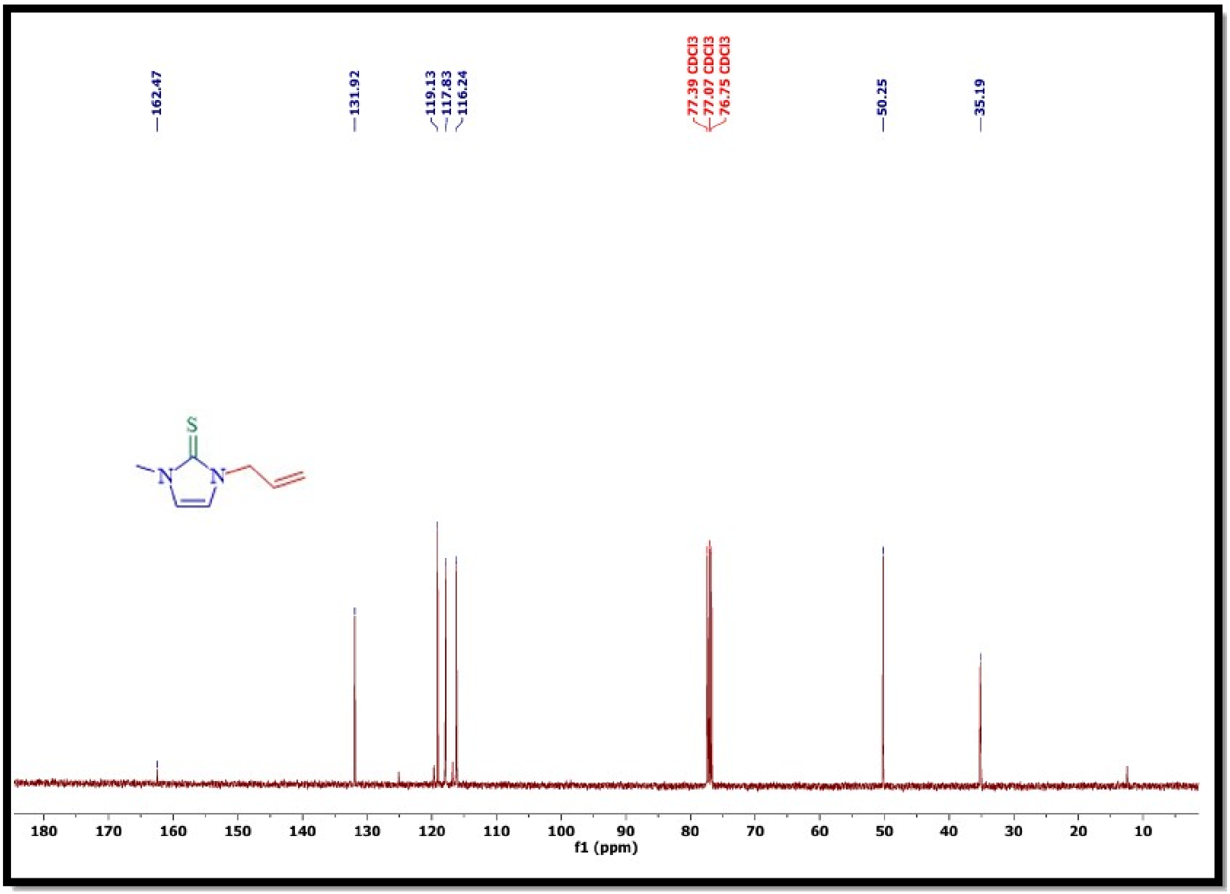

^{13}C-NMR of 1-allyl-3-methyl-1,3-dihydro-2H-imidazole-2-thione (Table 2, 3p).

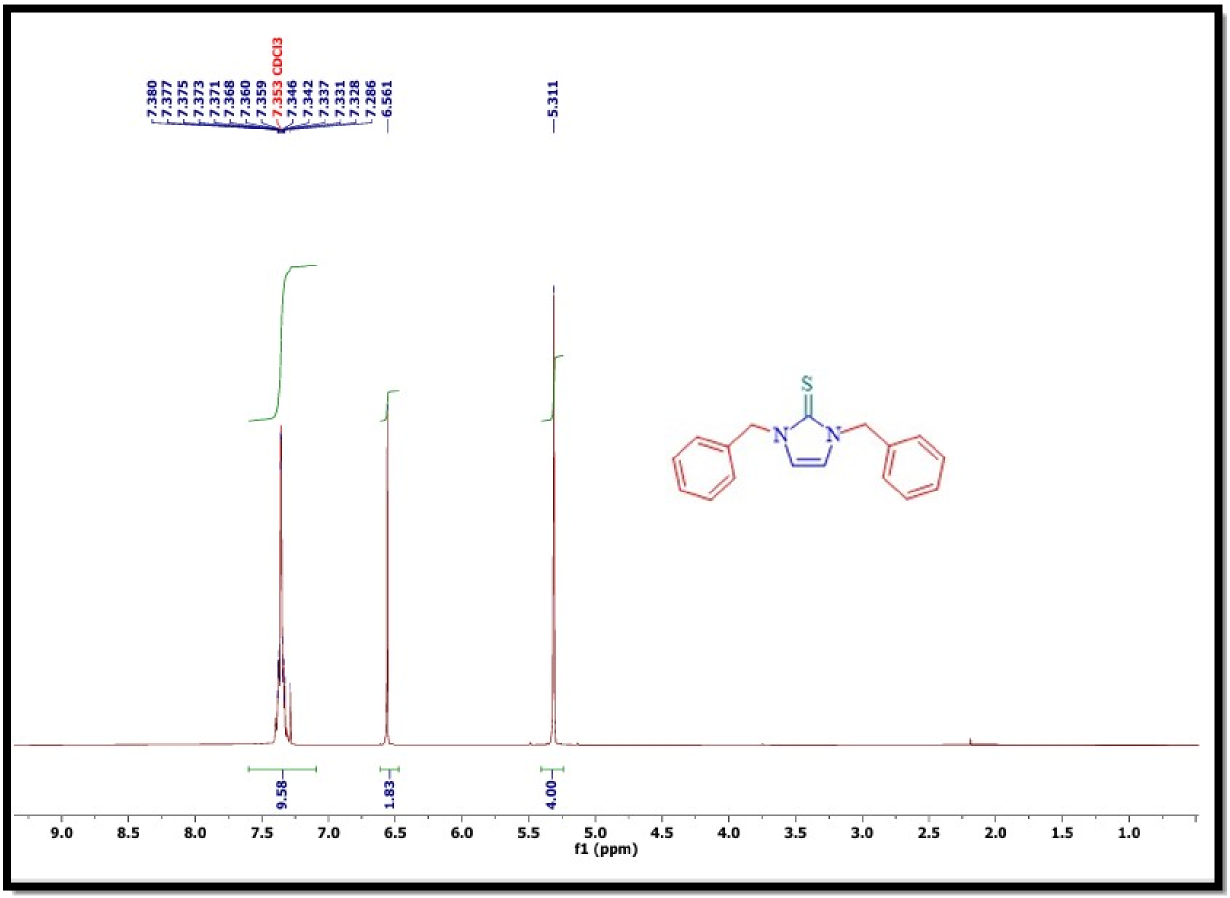

^{1}H-NMR of 1,3-dibenzyl-1,3-dihydro-2H-imidazole-2-thione (Table 2, 3q).

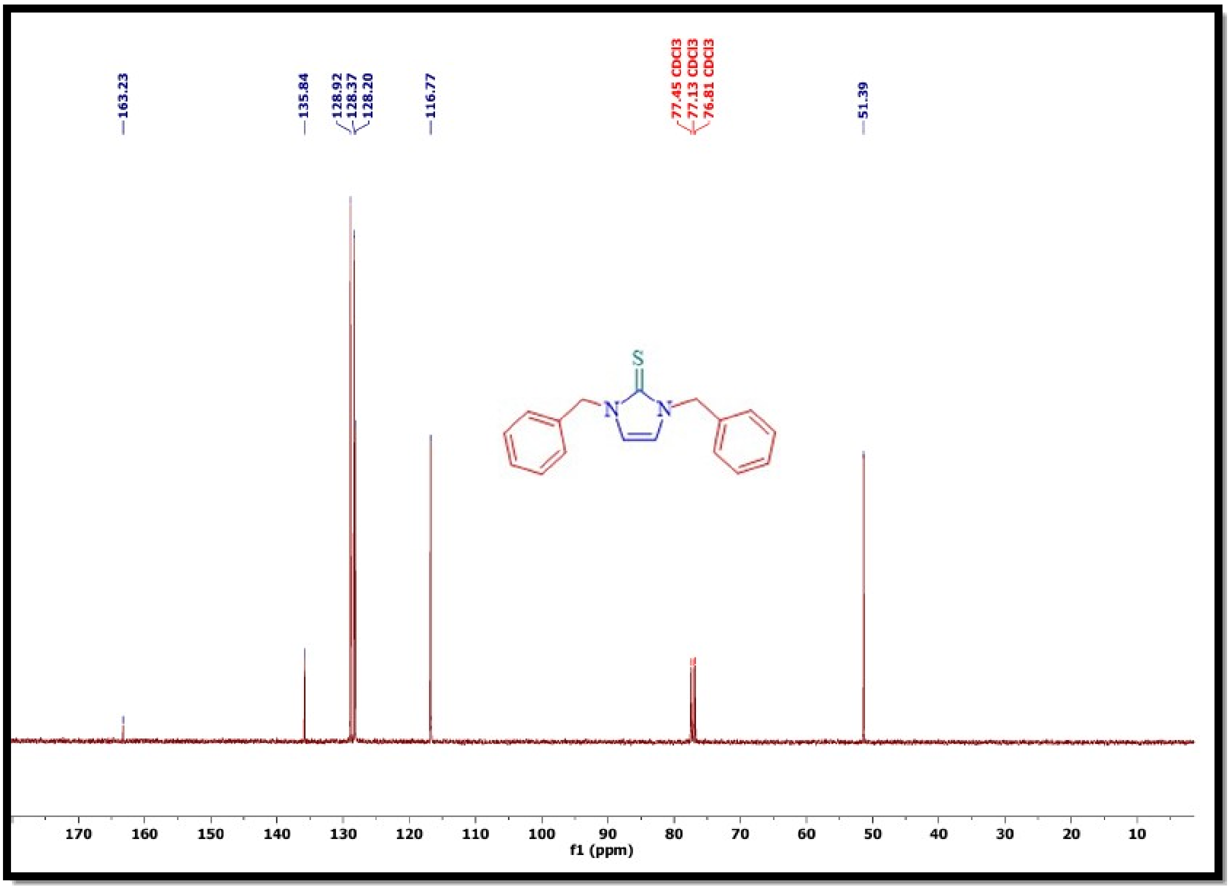

^{13}C-NMR of 1,3-dibenzyl-1,3-dihydro-2H-imidazole-2-thione (Table 2, 3q).

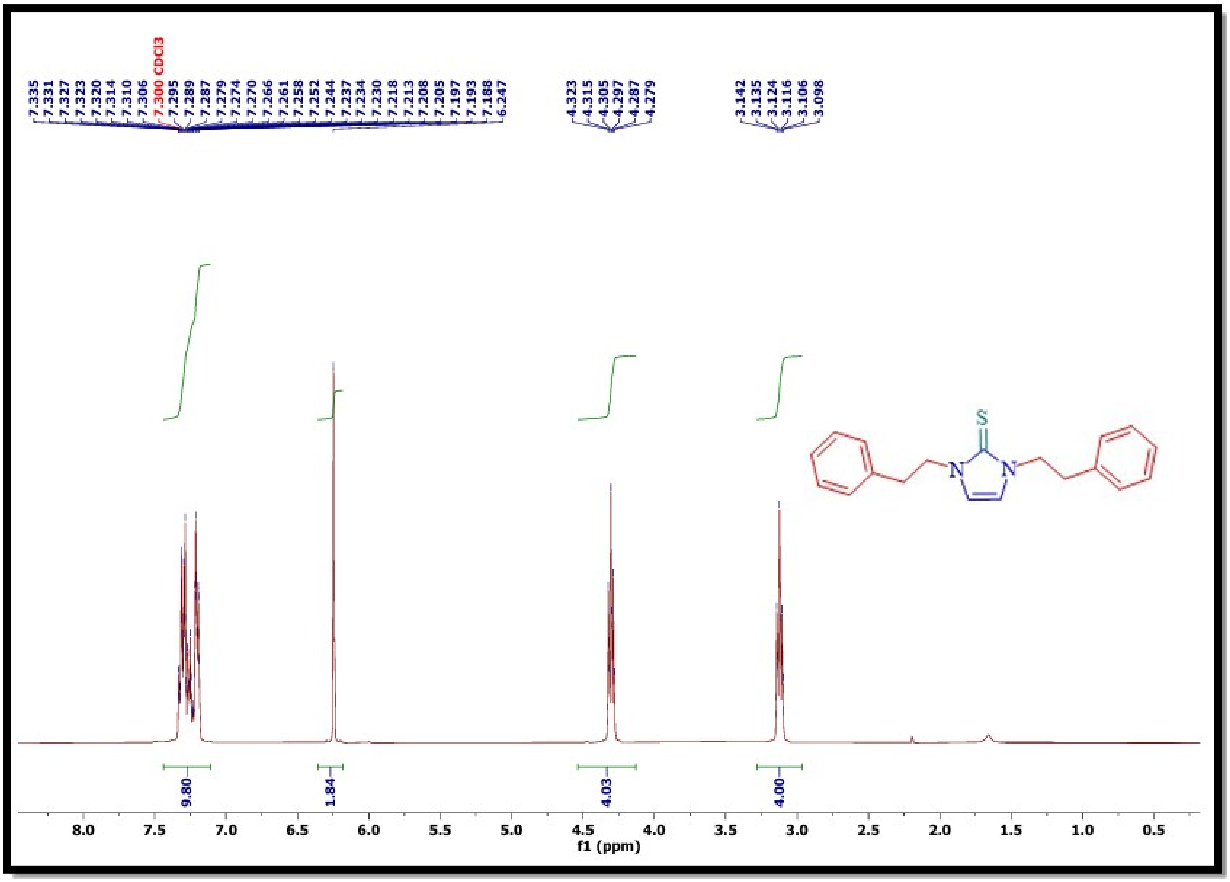

^{1}H-NMR of 1,3-diphenethyl-1,3-dihydro-2H-imidazole-2-thione (Table 2, 3r).

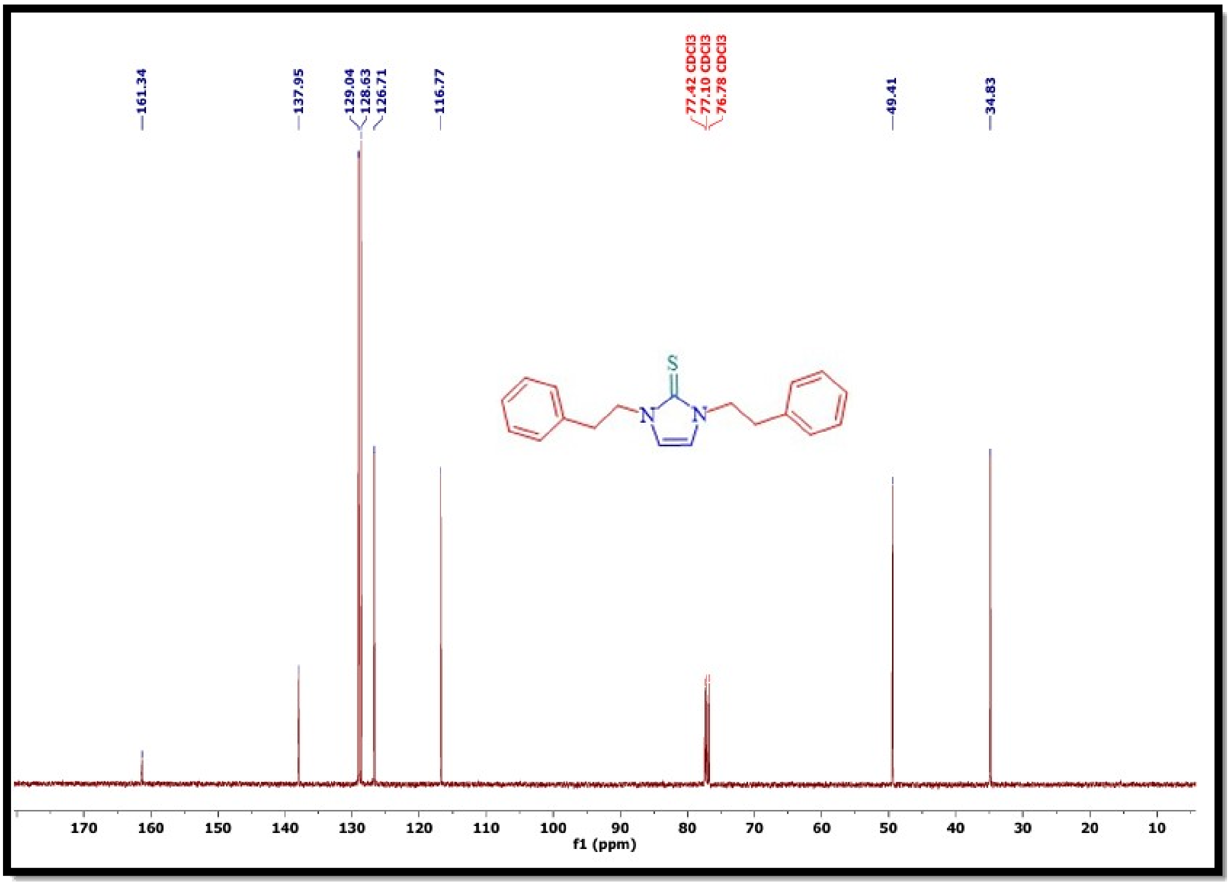

^{13}C-NMR of 1,3-diphenethyl-1,3-dihydro-2H-imidazole-2-thione (Table 2, 3r).

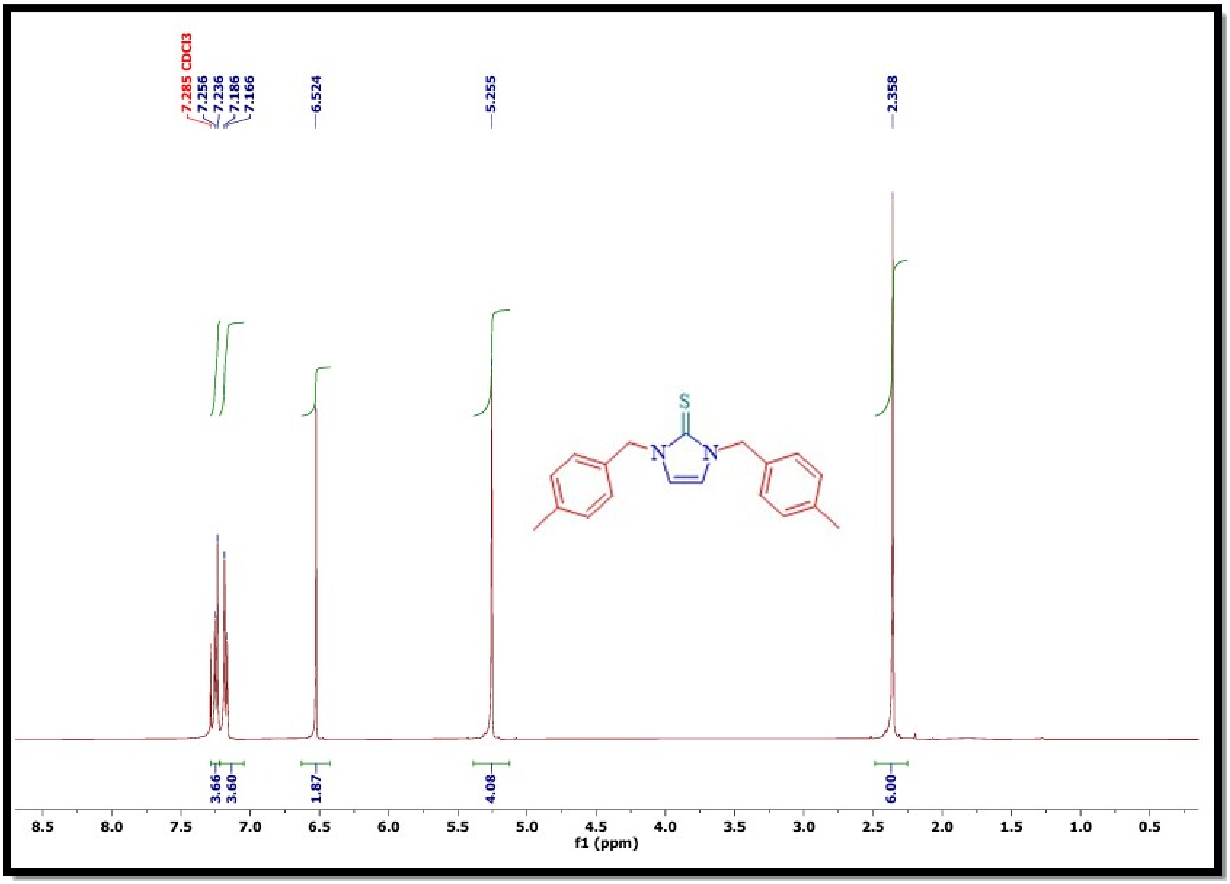

^{1}H-NMR of 1,3-bis(4-methylbenzyl)-1,3-dihydro-2H-imidazole-2-thione (Table 2, 3s).

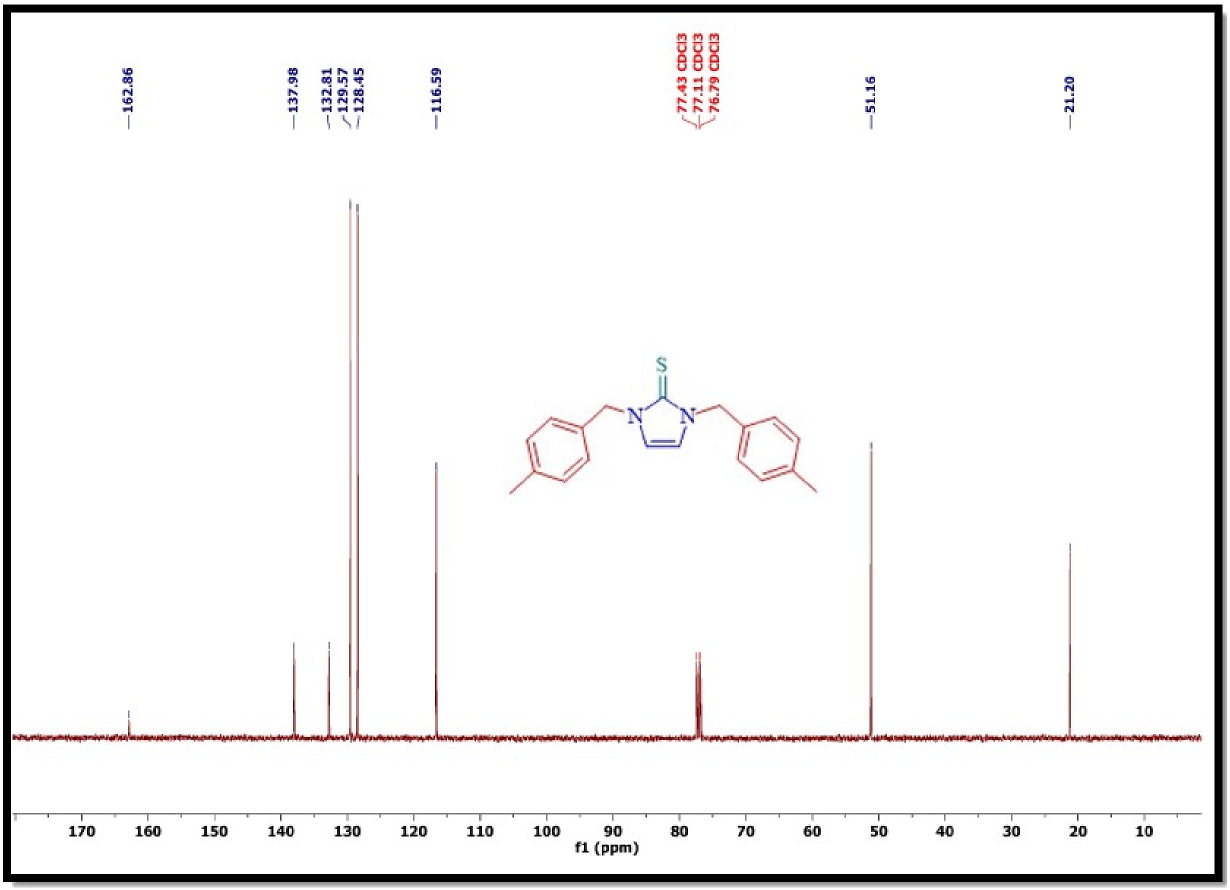

^{13}C-NMR of 1,3-bis(4-methylbenzyl)-1,3-dihydro-2H-imidazole-2-thione (Table 2, 3s).

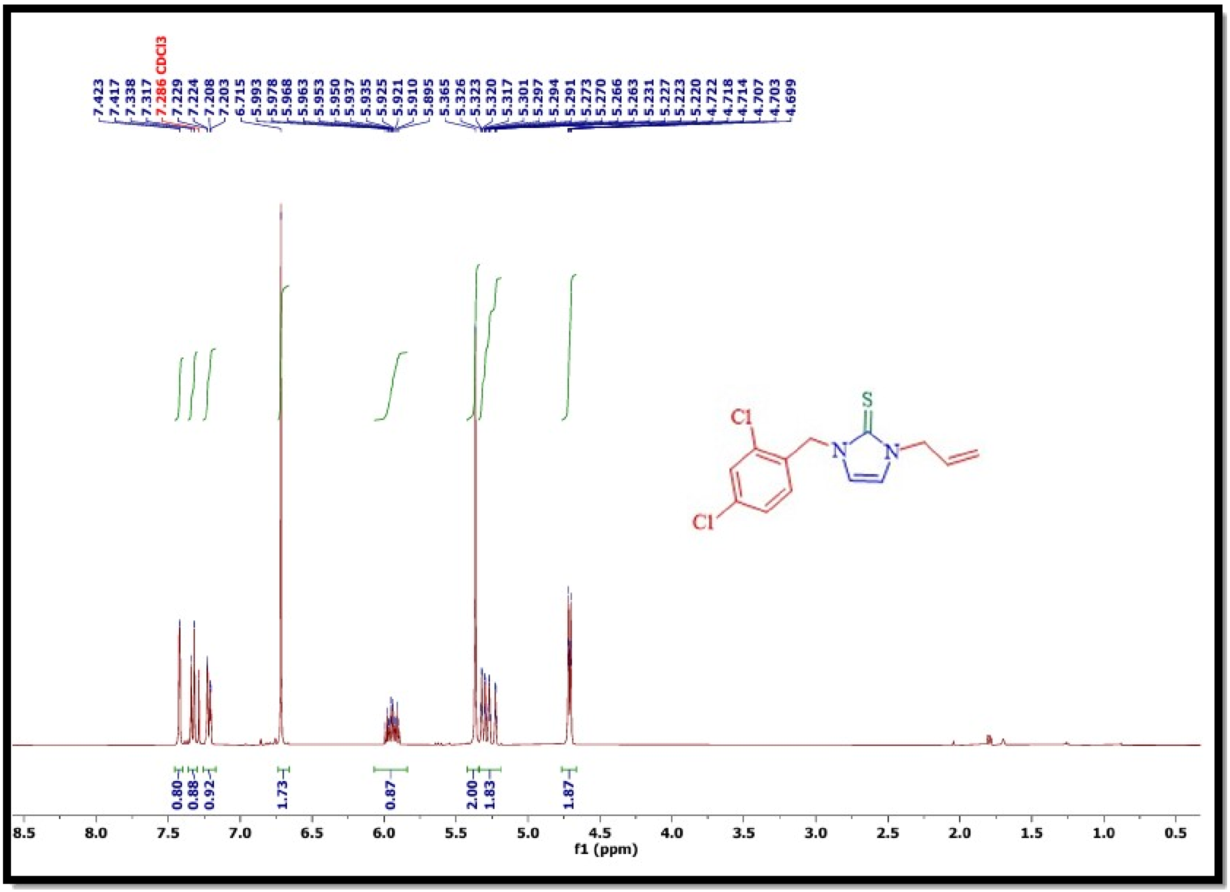

^{1}H-NMR of 1-allyl-3-(2,4-dichlorobenzyl)-1,3-dihydro-2H-imidazole-2-thione (Table 2, 3t).

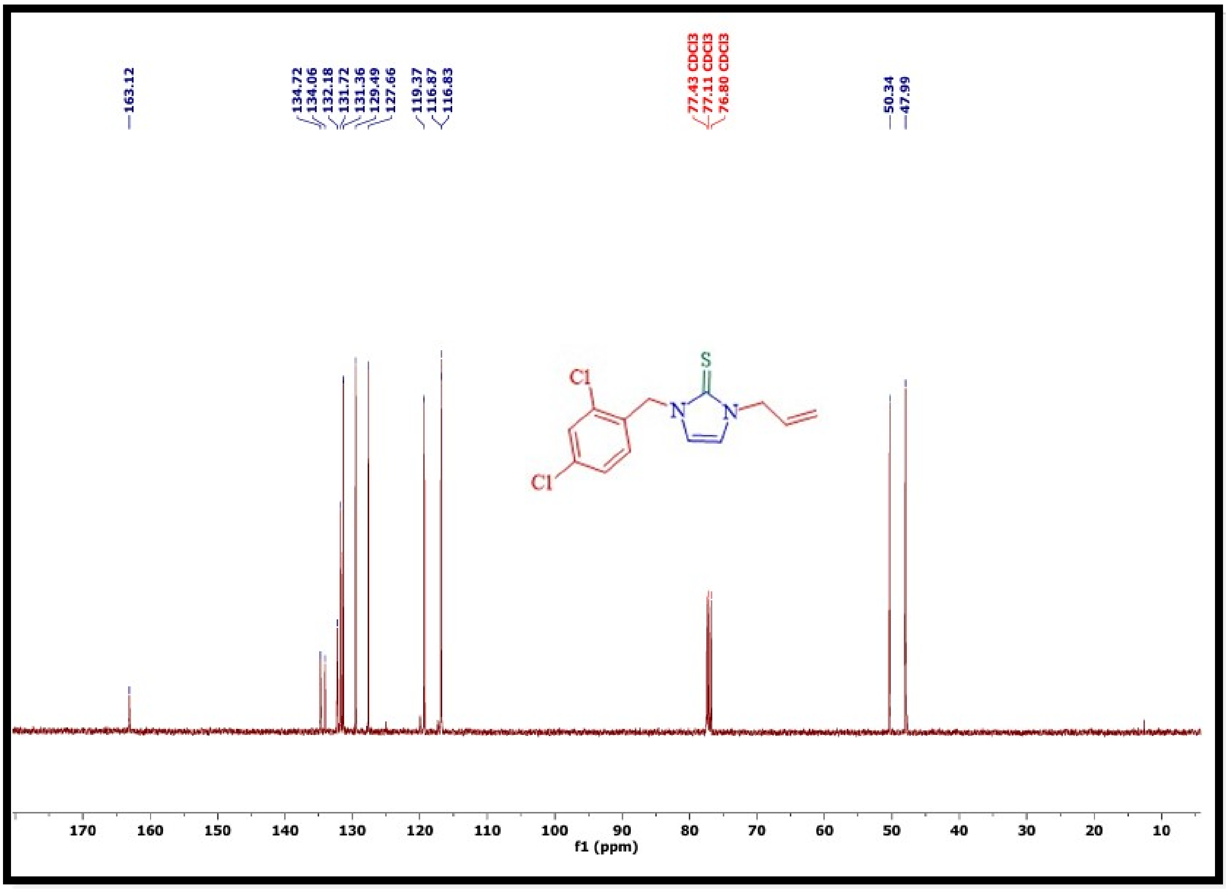

^{13}C-NMR of 1-allyl-3-(2,4-dichlorobenzyl)-1,3-dihydro-2H-imidazole-2-thione (Table 2, 3t).

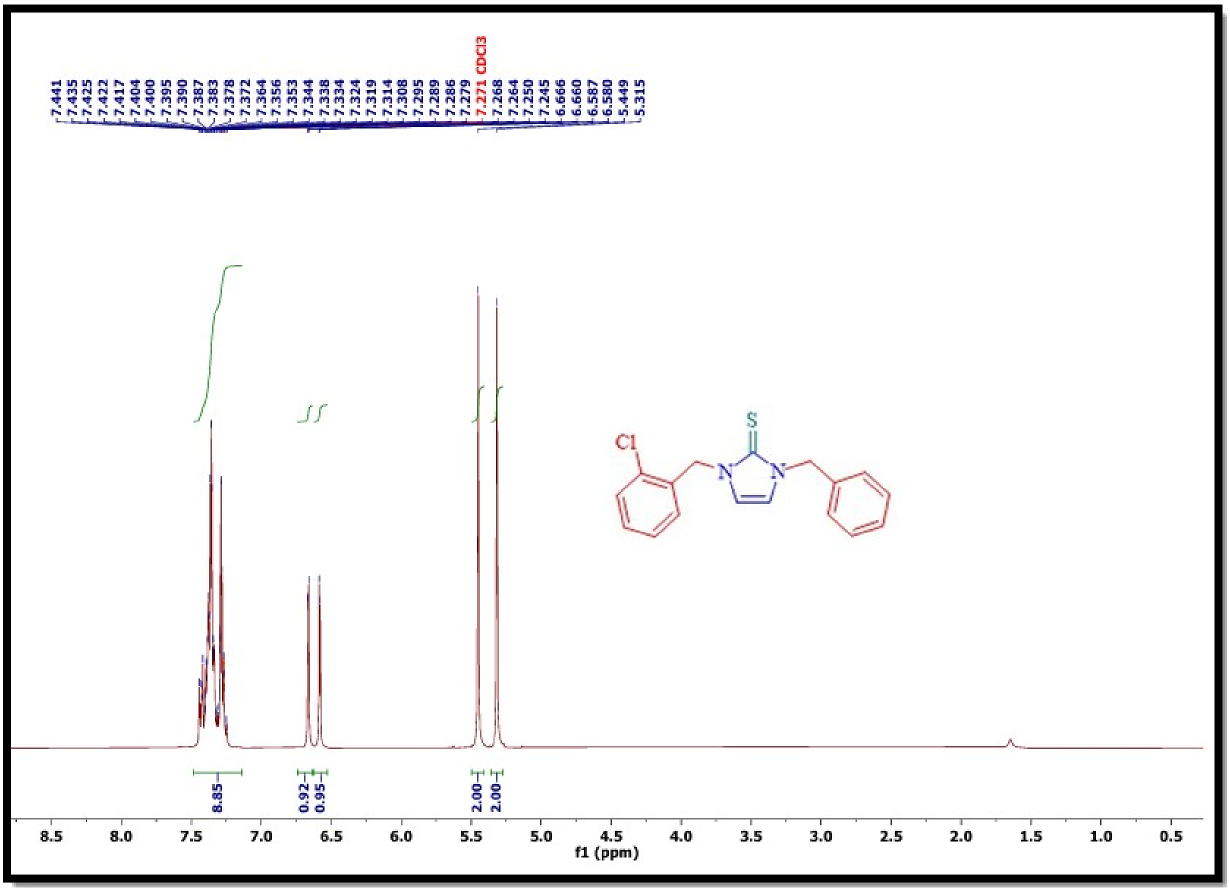

^{1}H-NMR of 1-benzyl-3-(2-chlorobenzyl)-1,3-dihydro-2H-imidazole-2-thione (Table 2, 3u).

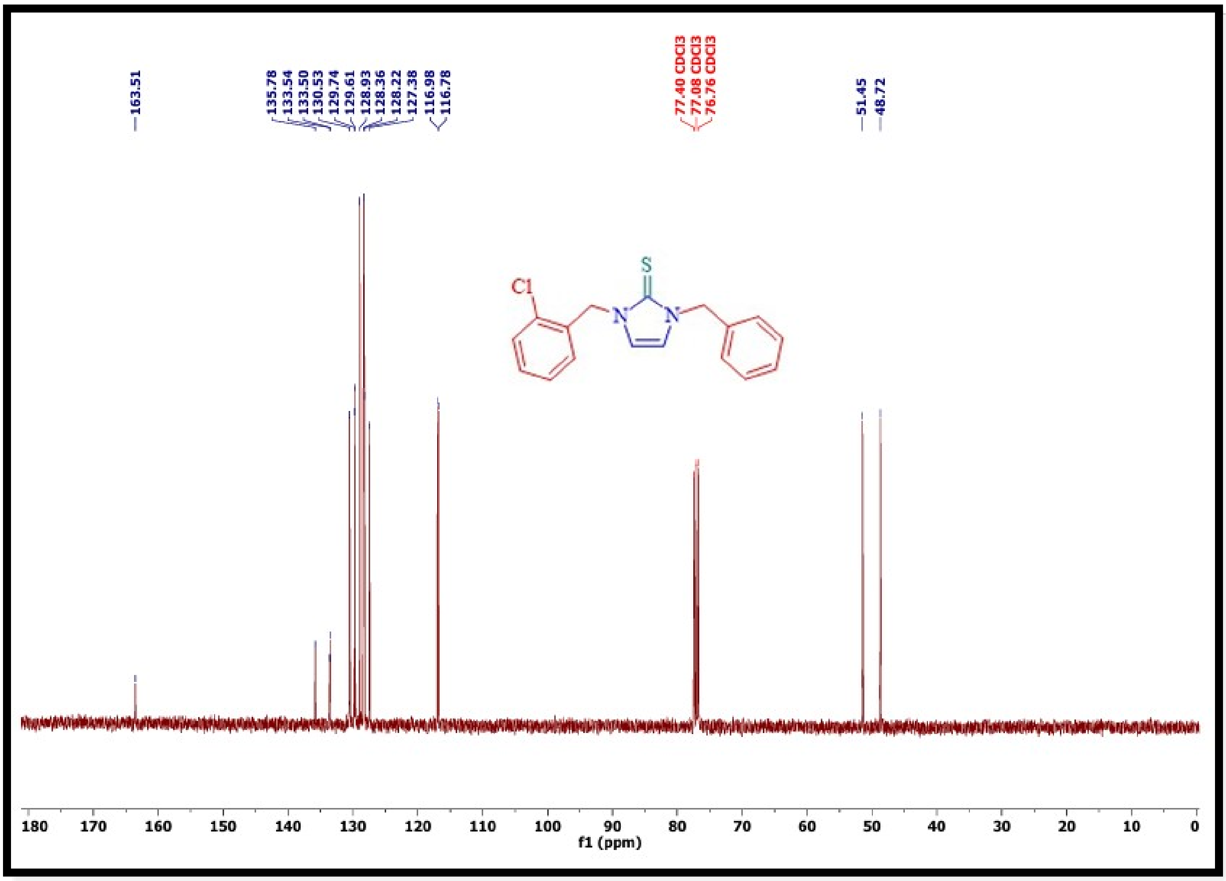

^{13}C-NMR of 1-benzyl-3-(2-chlorobenzyl)-1,3-dihydro-2H-imidazole-2-thione (Table 2, 3u).
